# *Selaginella bryopteris*-derived silver nanoparticles against ovarian cancer cells: green synthesis, *in vitro* anticancer activity, and exploratory *in silico* analysis

**DOI:** 10.3389/fphar.2026.1814120

**Published:** 2026-08-11

**Authors:** Sangita Dey, Khushbu Wadhwa, Neha Kumari, Chetan Pandey, Anoop Kumar, Vincenzo Calderone, Simone Brogi, Neha Kapoor

**Affiliations:** 1 Department of Biotechnology, North Bengal University, Darjeeling, West Bengal, India; 2 Fungal Biology Laboratory, Ramjas College, University of Delhi, Delhi, India; 3 Chemical Biology Lab, Hindu College, University of Delhi, Delhi, India; 4 Department of Pharmacy, University of Pisa, Pisa, Italy

**Keywords:** anticancer, nanotechnology, ovarian cancer, PARP protein, phytochemicals, silver nanoparticles (AgNPs)

## Abstract

**Introduction:**

Chronic hormonal imbalances and associated symptoms characterize ovarian cancer. PARP-1 inhibitors, such as olaparib and ruparib, are used to treat it, but new drugs are needed to overcome toxicity and resistance issues. PARP inhibition in *BRCA1/2*-mutated cancer cells leads to apoptosis.

**Methods:**

We synthesized silver nanoparticles (AgNPs) from *S. bryopteris* leaf extract, using it as a stabilizing and reducing agent. The extract was added to the AgNO_3_ solution and maintained at room temperature for 24 h. The AgNPs were characterized using various techniques, including UV-visible and FTIR spectroscopy, SEM, EDX, Transmission Electron Microscopy, and ICP-MS analysis. The stability of AgNPs was evaluated under different pH and temperature conditions, and their anticancer activity was evaluated against SKOV3 and OVCAR-3 ovarian cancer cells using MTT assay, while IOSE80 cells were used as a non-tumorigenic ovarian epithelial cell model. The binding mechanism of phytochemicals from *Selaginella bryopteris* to PARP-1 was investigated using computational methods.

**Results:**

*Selaginella bryopteris*-derived AgNPs showed stronger cytotoxicity than the plant extract against SKOV3 and OVCAR-3 ovarian cancer cells, with IC_50_ values of 9.56 and 10.45 μg/mL, respectively, and lower toxicity toward IOSE80 cells (IC_50_ = 19.75 μg/mL). AgNP treatment increased intracellular Reactive Oxygen Species levels, suggesting oxidative-stress-mediated cell death, while *in silico* analyses highlighted rhamnetin as a promising PARP-1-interacting phytoconstituent.

**Discussion:**

The anticancer properties of AgNPs from *S*. *bryopteris* were investigated using analytical, pharmacological, and computational methods. Results showed the stability of AgNPs and their effectiveness against ovarian cancer cells. AgNPs from the *S. bryopteris* leaf extract showed stronger anticancer activity than the plant extract alone, whereas their possible use as targeted drug delivery or controlled-release platforms remains a future perspective requiring dedicated experimental investigation.

## 1 Introduction

Ovarian cancer is one of the most prevalent cancers affecting women globally. It can disrupt the normal functioning of the female reproductive system, leading to a considerable decrease in the lifespan of women. The epidemiological trends of this cancer vary across geographic regions and change over time (Keyvani et al., 2023). Recently, ovarian cancer was identified as the seventh most common cancer in women worldwide (Shafabakhsh and Asemi, 2019). In India, ovarian cancer is next to cervical cancer and is the most common cancer of the female genital tract. According to GLOBOCAN 2022 estimates, ovarian cancer accounted for approximately 325,000 new cases and 207,000 deaths worldwide in 2022 (Filho et al., 2025). A malignant tumor in one or both ovaries in females is known as ovarian cancer. All ovarian cell types exhibit the potential to develop into ovarian cancer (Kroeger and Drapkin, 2017; Berek et al., 2018). The three primary types of ovarian cancer are germ cell tumors, sex-cord stroma tumors, and epithelial ovarian cancer (∼90%). The primary cause of ovarian cancer is the existence of mutations in genes associated with the mitogen activated protein kinase (MAPK) pathways, cyclin dependent kinase inhibitor 2A (CDKN2A), p-catenin genes, phosphatidylinositol-4,5-biphosphate 3-kinase, catalytic alpha subunit (PIK3CA), tumor protein 53 (TP53), and v-Raf murine sarcoma viral oncogene homolog B (BRAF). In addition, ovarian cancer is characterized by a lack of tumor suppression gene expression due to AT-rich interaction domain 1A (*ARIDIA*) gene mutations (Rojas et al., 2016). Moreover, *ERB2* gene amplification that codes for the human epidermal growth factor receptor 2 (HER-2) is also observed in patients with ovarian cancer. The mode of ovarian cancer spread is distinct from that of other cancers due to the extremely rare occurrence of hematogenous metastasis. Ovarian cancer spreads mainly through passive diffusion, where cancer cells shed from the tumor and carried by peritoneal fluid movement. This process leads to the accumulation of pathologic fluid and formation of ascites, which facilitates the further spread of cancer cells (Yeung et al., 2015). Various treatments, including surgery, chemotherapy, radiation, hormonal, and phototherapy, are available for the management of ovarian cancer (Cortez et al., 2017; Summer et al., 2024c). Chemotherapy, which is widely used in cancer treatment, can have severe undesired effects, and patients undergoing chemotherapy may develop multiple side effects and drug resistance along with a high risk of recurrence (Yin et al., 2022). Chemotherapeutic agents efficiently kill dividing cancer cells; however, most available drugs cannot distinguish between cancerous and non-cancerous cells, thereby harming healthy cells and targeting undesired cellular targets (Pearce et al., 2017).

Conventional therapies have limitations, including the potential side effect of causing inflammation due to the inability of the drug to penetrate malignant tissues (Summer et al., 2024c). In contrast to this, targeted therapies are directed toward cancer-specific mutations and inhibit tumor growth. This minimizes the negative effect on nearby non-malignant cells (Torino et al., 2013; Anand et al., 2023). Targeted therapies offer better outcomes and fewer side effects. They aim to overcome the challenges of conventional therapy by specifically targeting cancerous cells (Summer et al., 2024c).

Furthermore, nanotechnology-based therapeutic and diagnostic approaches have been shown to improve cancer therapy (Ratan et al., 2020). Nanotechnology has received attention owing to its distinctive properties and broad-range of applications in agriculture, electronics, and medicine (Mohammad et al., 2022). Among the various available metallic nanoparticles (NPs), silver nanoparticles (AgNPs) are a highly commercialized choice worldwide because of their physicochemical and biological characteristics (Summer et al., 2024a). NPs usually range in size from 1 to 100 nm and exhibit unique physical and chemical properties. The biocompatibility of NPs depends on their size, and their surface reactivity increases with decrease in size. Smaller NPs may more efficiently interact with and enter cells due to their higher surface area-to-volume ratio; however, their biological effects depend on multiple parameters, including size, surface chemistry, concentration, and exposure conditions (Summer et al., 2024b).

AgNPs can be synthesized by various methods, including chemical reduction, physical vapor deposition, electrochemical methods, and green or biological synthesis (Iravani et al., 2014). The biological or green synthesis of AgNPs can be achieved by using bacteria, fungi, algae, and plant parts. This method has several advantages, including reduced toxic byproducts, enhanced biocompatibility, and lower costs (Gericke and Pinches, 2006; Mandal et al., 2006).

The green synthesis of AgNPs from plant extracts minimizes the use of hazardous chemical agents and makes them more ecofriendly methods as compared with conventional physical and chemical processes (Zulfiqar et al., 2024). Abbas et al. reported the radical scavenging activity of zinc oxide NPs synthesized from *Bauhinia variegata* extracts, highlighting the role of phytocompounds, including polyphenols, tannins, and flavonoids, in capping and stabilizing these NPs (Abbas et al., 2024). Although NPs have shown significant potential as anticancer and antiangiogenic agents, their possible risks should be carefully considered, as their biological effects may depend on size, morphology, surface chemistry, dose, and exposure conditions. These concerns may be mitigated through safer synthesis approaches, surface modification, optimization of NPs shape and structure, and encapsulation into suitable drug delivery systems (Mishra and Singh, 2015; Choi et al., 2016). Despite the potential risks associated with NPs, these issues may be mitigated through safer synthesis approaches, including biological synthesis (Wadhwa et al., 2025), surface modification to improve biocompatibility (Chung et al., 2008), optimization of NPs shape and structure (Manikandan et al., 2026), and encapsulation into drug delivery systems to reduce toxicity and improve safety (Thanigaivel et al., 2021). Several phytochemicals derived from medicinal plants, including curcumin, quercetin, resveratrol, and silymarin, are generally used to treat different types of cancers by inhibiting angiogenesis and apoptotic pathways. In a recent study, researchers found that silymarin loaded Ni-Fe metal-organic frameworks have anticancer activity against breast and neuroblastoma cancer cells (Rahimi et al., 2024). They also synthesized nanosized metal-organic frameworks (MOFs) using phytic acid and Fe^3+^ to deliver mitoxantrone, which was tested against metastatic bone tumors (Huang et al., 2023). Natural products are a major source of anticancer compounds that inhibit cancer cell proliferation. Elemene, a sesquiterpene from *Curcuma wenyujin*, is linked to the antitumor compound nitric oxide. Elemene-nitric oxide hybrids were tested against lung, glioblastoma, and colorectal cancer cell lines (Bai et al., 2022). Xanthohumol, a compound found in hops, has anticancer effects on gastric cancer cells. It reduces cell viability by inhibiting cell growth and inducing programmed cell death (Wei et al., 2018). The antibacterial activity of *Selaginella bryopteris*-derived AgNPs against *M. luteus*, *P. mirabilis*, *B. megaterium*, and *E. coli* was evaluated using disc diffusion and agar well diffusion methods (Yadav et al., 2020), and antifungal activity against *Candida spp*. (Wadhwa et al., 2025). Although the green synthesis of AgNPs has been widely investigated, the anticancer potential of *S. bryopteris*-derived AgNPs against ovarian cancer cells remains poorly explored. In particular, previous studies mainly focused on the antimicrobial and antifungal properties of *S. bryopteris*-based AgNPs, whereas an integrated evaluation combining AgNPs characterization, ovarian cancer cytotoxicity, ROS-mediated effects, and PARP-1-oriented computational analysis has not been reported. Accordingly, in this study, AgNPs were synthesized with AgNO_3_ using aqueous *S*. *bryopteris* leaf extracts, following a previously developed method (Yadav et al., 2020; Wadhwa et al., 2023b; Wadhwa et al., 2025), and tested against SKOV3 and OVCAR-3 ovarian cancer cell lines and the non-tumorigenic ovarian epithelial cell line IOSE80. The rationale of the present study is based on an integrated framework linking the phytochemical composition of *S. bryopteris*, green synthesis of AgNPs, biological evaluation, and computational analysis. Phytoconstituents present in the leaf extract may act as reducing, capping, and stabilizing agents during AgNP formation and may also contribute to the biological properties of the resulting NPs system. Therefore, after AgNP synthesis and physicochemical characterization, the anticancer activity of the green-synthesized AgNPs was evaluated in ovarian cancer cells. In parallel, selected phytocompounds identified in the present GC-MS analysis or previously reported for *S. bryopteris* and related *Selaginella* species were explored by *in silico* analysis to generate hypotheses on their potential interaction with PARP-1. This integrated approach was intended to connect plant-derived phytochemistry, NPs formation, *in vitro* anticancer activity, and computational target-oriented investigation.

### 1.1 Selaginella bryopteris

**FIGURE 1:**
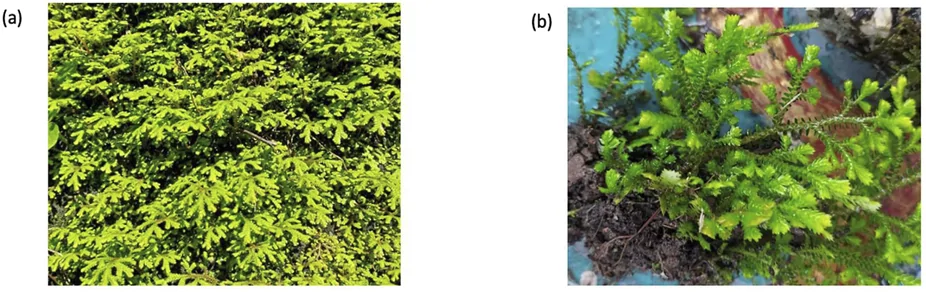
**(a)** *Selaginella bryopteris* in its natural habitat and **(b)** Plant morphology.

*Selaginella bryopteris* (L.) Bak or resurrection plant is a desiccation-tolerant medicinal plant belonging to the Selaginellaceae family (Pandey et al., 2010). It is also known as “Sanjeevani booti” for its therapeutic and medicinal properties. The herb is mentioned in historical epics, such as the Ramayana. *Selaginella bryopteris* is a perennial, herbaceous plant that grows in humid environments on rocks. It has microphyll leaves and ligules, with roots and spore-bearing structures called sporangia in the axils of sporophylls (Antony and Thomas, 2011). During summer, this plant undergoes extreme desiccation, and its fronds become curled and dry. When the monsoon arrives, *Selaginella* turns green and returns to its active life (Sah et al., 2005). The plant is also an example of an unspecialized primitive heterospory (Schulz et al., 2010) and plays a key role in evolutionary studies showing potent adaptation to xeric conditions (Figure 1).

*Selaginella* contains several types of secondary metabolites, including flavonoids, phenols, and alkaloids, which play a vital role in defense mechanisms. Secondary metabolites from the plant act as reducing and stabilizing agents for NPs synthesis. The plant has been used to treat fever, kidney problems, asthma, and diarrhea since ancient times. Ethanolic *S. bryopteris* extracts exhibit significant wound healing properties (Paswan et al., 2020). GC-MS analysis indicated the presence of flavonoids, bioflavonoids, sugar alcohols, glycosides, campesterol, stigmasterol and β-sitosterol (Paswan et al., 2020; Gautam et al., 2023).

### 1.2 PARP-1 and associated single-strand break (SSB) repair

**FIGURE 2:**
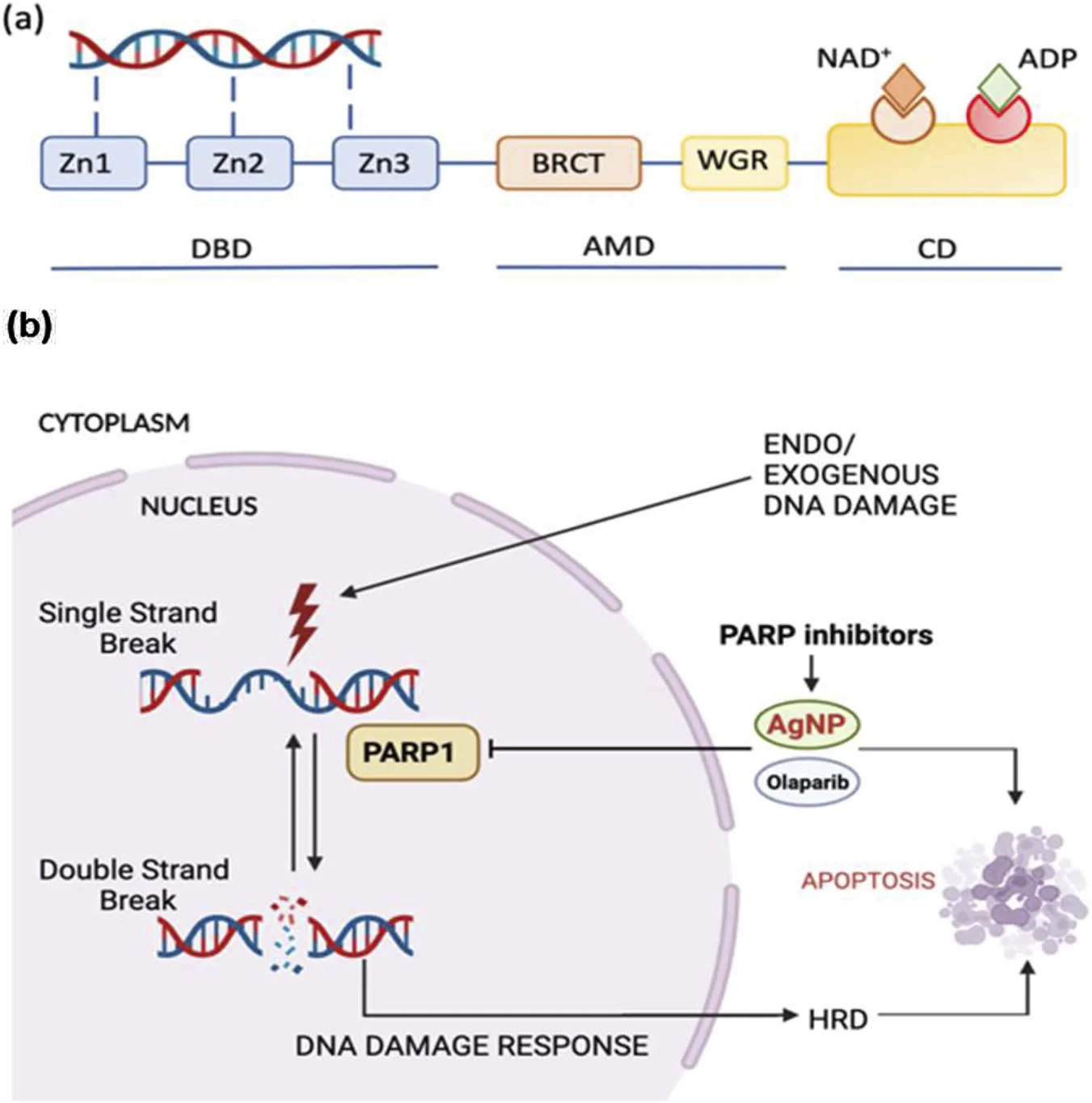
**(a)** Schematic representation of various functional domains of the PARP-1 protein, including the DNA-binding domain (DBD), auto-modification domain (AMD), and catalytic domain (CD). **(b)** Proposed mode of action of PARP-1 inhibitors (AgNPs and olaparib) against exo/endogenous DNA damage in cancer cells.

The PARP family of proteins has attracted attention for cancer treatment. They are also known as ADP-ribosyl transferase diphtheria-toxin-like proteins (ARTD1–17). In humans, 17 PARP proteins exist, each with a common catalytic domain (Lu et al., 2019). However, only four of these enzymes (PARP-1, 2, 5A, and 5B) can form long poly-ADP-ribose chains and attach them to their target molecules (Burkle and Virag, 2013). The PARP proteins play key roles in DNA repair, transcription, replication, and chromatin structure (Figure 2a). They also support centrosome function, control telomere dynamics, facilitate endosomal movement, and influence apoptosis-mediated cell death (Burkle, 2005).

The PARP proteins play a crucial role in DNA repair pathways, particularly in base excision repair (BER) (Chen and Du, 2018). BER maintains DNA integrity during oxidative stress induced by toxic chemicals or mutations. Impaired BER or PARP can lead to single-strand breaks (SSBs), which can accumulate and cause double-strand breaks (DSBs). This can alter the mechanisms of homologous recombination (HR) and non-homologous end joining (NHEJ), making cells more susceptible to cancer progression in susceptible individuals with defective HR and NHEJ cells (Maynard et al., 2009).

In scientific research, the primary PARP family members are PARP-1 and PARP5A (Kleine et al., 2008). The most common protein, PARP-1, is found in the nucleus and plays a major role in DNA repair when DNA damage occurs. This enzyme is an ADP ribosyl transferase that catalyzes the synthesis of poly-ADP-ribose from NAD^+^ molecules, leading to chromatin reorganization and DNA repair protein recruitment. Understanding molecular interactions is crucial for designing PARP-1 inhibitors (Rojo et al., 2012). The PARP-1 protein has three zinc DNA-binding domains at its N-terminal region, which helps it recognize DNA breaks. The second domain, the auto-modification domain (AMD) contains a tryptophan-glycine-arginine (WGR)-rich domain and a BRCA1 C-Terminus (BRCT) domain (Figure 2b) (Langelier and Pascal, 2013; Nilov et al., 2020). The catalytic domain of PARP-1 protein has two binding sites: the donor (NAD^+^) and acceptor (PAR) binding sites. NAD^+^ binds to PARP-1 by forming two hydrogen bonds with Gly863 and hydrophobic contacts with Tyr907 (π-π stacking). Thus, ADP forms hydrophobic contacts with Met890 along with hydrogen bonds with Lys903 and Glu988 residues (Nilov et al., 2020).

Several PARP inhibitors have been approved for the treatment of selected cancers, including ovarian cancer, particularly in tumors with BRCA mutations or impaired homologous recombination repair. Their therapeutic rationale is based on blocking PARP-mediated DNA repair and exploiting pre-existing DNA repair vulnerabilities in cancer cells, thereby promoting the accumulation of DNA damage and cancer cell death. Among them, Niraparib, olaparib, and talazoparib, are clinically available PARP inhibitors, although their use is associated with relevant adverse effects, including fatigue, elevated cholesterol, headaches, dizziness, nausea, vomiting, and renal toxicities. In ovarian cancer, PARP inhibitors are used both as first-line maintenance therapy in advanced disease and in recurrent platinum-sensitive settings. Accordingly, there remains a pressing need to develop novel, selective PARP-1 inhibitors with improved safety profiles and reduced undesired effects (LaFargue et al., 2019).

## 2 Materials and methods

### 2.1 Materials

Dried *S*. *bryopteris* was procured from the local market and authenticated by a taxonomist at the Department of Botany, University of Delhi. Human ovarian carcinoma OVCAR-3 cells were obtained from the National Centre for Cell Science (NCCS), Pune, India. Human ovarian carcinoma SKOV3 cells and normal human ovarian surface epithelial IOSE80 cells were kindly provided by the Indian Institute of Chemical Biology (IICB), Kolkata, India. AgNO_3_ was obtained from Sigma-Aldrich (St. Louis, MO, United States).

### 2.2 Collection and processing of leaf extract

The dried *S. bryopteris* plant material was carefully inspected and cleaned with double-distilled water to remove dust, debris, and unwanted material. The cleaned material was then re-dried at room temperature to remove residual moisture and powdered using a grinder. For extract preparation, 1.25 g of the dried powdered material was mixed with 25 mL of distilled water and stirred for 1 h at room temperature using a magnetic stirrer. The aqueous leaf extract was filtered using Whatman filter paper, and the filtrate was centrifuged at 4,700 rpm for 10 min to remove any remaining residues. The obtained aqueous leaf extract was carefully covered with aluminum foil and stored at 4 °C until use for AgNP synthesis. The resulting leaf extract was used as a reducing, capping, and stabilizing agent for the green synthesis of AgNPs.

### 2.3 Physicochemical characterization of leaf extract of *Selaginella bryopteris*

#### 2.3.1 Gas chromatography-mass spectrometry (GC-MS) analysis

Sample was injected into an GC-MS QP2010 model (Shimadzu®), Column, GC, SH-I-5Sil MS Capillary, 30 m × 0.25 mm × 0.25 um, injection mode: Splitless. The operating conditions of the GC–MS set for the analysis were as follows: oven temperature 45 °C for 2 min then 140 °C at 5 °C/min and finally increased to 280 °C and held isothermally for 10 min. The sample injection was 2 μL and the carrier gas was helium at 1 mL/min. The ionization of the sample components was carried out 70 eV. The running time of the GC was from 9.10 min–52.0 min. NIST14. L library (2020) was then searched to compare the structures of the compounds with that of the NIST database. Compounds were then identified based on the retention times and mass spectra with already known compounds in the NIST library (C:\Database\NIST20.L).

#### 2.3.2 ICP-MS (inductively coupled plasma-mass spectrometry) based quantification of constituents of leaf extract of *Selaginella bryopteris*

The chemical composition of the *S. bryopteris* leaf extract was determined by ICP-MS analysis after microwave digestion. The dried powdered leaf extract was placed in a clean Teflon microwave digestion vessel with 2 mL of concentrated nitric acid and 6 mL of concentrated hydrochloric acid. Digestion was performed using a scientific microwave at 165 °C for 10 min with irradiation at 1000 W for 15 min. After cooling, the sample was analyzed using an Agilent 8900 Triple Quadrupole ICP-MS (model number G3665A).

### 2.4 Green synthesis of AgNPs using leaf extract of *Selaginella bryopteris*

For AgNP synthesis, 45 mL of freshly prepared 1 mM AgNO_3_ aqueous solution was placed in a dark amber bottle to prevent photo-oxidation and then mixed with 5 mL of *S. bryopteris* leaf extract under magnetic stirring- The AgNPs suspension was maintained at room temperature for 24 h at pH 7 and then analyzed by using UV-visible spectroscopy (Yadav et al., 2020). After 48 h, the AgNPs suspension was centrifuged at 12,000 rpm for 15 min and washed at least three times with double distilled water to remove unbound leaf extract. The color change from transparent to red brown indicated the formation of AgNPs.

### 2.5 Characterization of green synthesized AgNPs

#### 2.5.1 UV–visible absorption spectroscopy

To monitor AgNP formation, a 2 mL aliquot of the reaction mixture was collected after 24 h and analyzed by UV–visible absorption spectroscopy using a UV–visible spectrophotometer (UV-1800, Shimadzu, Japan). Spectra were recorded over the wavelength range of 200–800 nm. To avoid signal saturation in the UV region, 300 µL of the *S. bryopteris* leaf extract stock solution (50 mg/mL) was diluted with 3.0 mL of double-distilled water to a final volume of 3.3 mL, corresponding to an 11-fold dilution and a final extract concentration of approximately 4.55 mg/mL. The diluted extract was then analyzed by UV–visible spectroscopy. The spectra were used to evaluate the reduction of silver ions and the appearance of the characteristic surface plasmon resonance (SPR) band of AgNPs.

#### 2.5.2 Morphological analysis using field emission scanning electron microscopy (FESEM) and energy dispersive X-ray (EDX)

The surface morphology and size of the AgNPs were determined using field emission scanning electron microscopy (FESEM) with a Zeiss GeminiSEM 500 system (Germany) equipped with an energy-dispersive X-ray spectroscopy (EDX) detector for elemental analysis. Prior to analysis, the dried AgNPs were redispersed in double-distilled water and sonicated for sufficient time to obtain a uniform dispersion. A small amount of the sonicated sample was drop-cast on a platinum grid and air-dried. The prepared samples were analyzed by FESEM to evaluate AgNP shape, morphology, and surface texture, while EDX analysis was used to assess elemental composition.

#### 2.5.3 Transmission electron microscopy (TEM)

Transmission Electron Microscopy (TEM) was used to determine the nanoscale size and morphology of the AgNPs. The sonicated samples were dropped onto a carbon-coated copper grid, allowed to dry at room temperature, and then analyzed using a TALOS L120C transmission electron microscope (Thermo Fisher Scientific).

#### 2.5.4 FTIR spectroscopy for biomolecular characterization

FTIR spectroscopy was conducted to identify the functional group present in *S. bryopteris* leaf extracts that may be responsible for the reduction and stabilization of AgNPs during biosynthesis. A Perkin Elmer spectrophotometer operating in transmission mode within the range of 400–4,000 cm^−1^ at a resolution of 4 cm^−1^ was used to generate the FTIR spectra.

#### 2.5.5 X-ray diffraction (XRD) studies

The crystalline nature of the green-synthesized AgNPs was evaluated using a high-resolution X-ray diffractometer (Rigaku SmartLab SE, Tokyo, Japan) equipped with a Cu X-ray source operating at 3 kW. The analysis was performed over a 2θ range of 10°–80°. The obtained diffraction patterns were compared with standard powder diffraction data from the Joint Committee on Powder Diffraction Standards (JCPDS).

#### 2.5.6 Dynamic light scattering (DLS) and zeta potential analysis

The hydrodynamic diameter and zeta potential of the green-synthesized AgNPs were determined using a Zetasizer instrument (Malvern, UK), with distilled water used as the dispersant medium. Briefly, the AgNP stock solution was diluted 10-fold prior to analysis to determine particle size distribution, surface charge, and electrostatic stability (Wadhwa et al., 2025).

#### 2.5.7 Thermogravimetric (TGA) and derivative thermogravimetric (DTG) thermal analysis

TGA was performed by using TA TGA 5500 to determine the thermal stability and composition of AgNPs. Tru-MassTM, a thermomicrobalance was used to measure the change of mass of sample, i.e., AgNPs as function of temperature under the controlled environment of heating rate 10 °C/min by using nitrogen gas atmosphere.

#### 2.5.8 ICP-MS analysis of green synthesized AgNPs

To determine the amount of silver content in green synthesized AgNPs, ICP-MS (ICP-MS system, Agilent 7900). ICP ionizes the atoms of the elements present in a sample and then these ions are separated and detected by the mass spectrometer. For ICP-MS analysis, samples were digested with acid by using 1% nitric acid (HNO_3_) (analytical quality cc. HNO_3_ in Milli-Q water). After digestion with acid, argon discharge, with a temperature range of 6,000–10,000 K was used to ionize the sample and then detected *via* a mass spectrometer having orthogonal detector system (ODS).

### 2.6 Stability of the AgNPs at different pH and temperature conditions

The pH stability of green synthesized AgNPs was investigated by changing the pH of AgNPs. For this, pH levels of 3, 5, 7, 8, and 9 were used. The pH of the solution was maintained using freshly prepared 10% HNO_3_ and 0.1 M NaOH (Muhammad Tahir et al., 2020; Mumtaz et al., 2023; Summer et al., 2023). The heat stability of the AgNPs was checked at different temperatures, such as 3 °C, 55 °C, and 70 °C, and at room temperature (stock AgNPs). After 24 h of incubation, the absorption spectra of the reaction mixture were observed using a UV-visible spectrophotometer. The samples were thoroughly mixed and vortexed before UV-visible spectral acquisition. For these analyses, AgNPs were dispersed and redispersed in double-distilled water. Each sample was prepared in triplicate, and the resulting data were plotted.

### 2.7 *In silico* studies

#### 2.7.1 Molecular dynamics on AgNPs

The Desmond software used Maestro 12.6 as its graphical interface to set the molecular dynamics simulation parameters (Desmond Molecular Dynamics System 6.4 academic version, D. E. Shaw Research (“DESRES”), New York, NY, United States, 2020. Maestro-Desmond Interoperability Tools, Schrödinger, New York, NY, United States, 2020). The Desmond software system builder function was used to produce the AgNP system. The AgNP model used for the simulation consisted of an atomistic silver cluster composed of 236 Ag atoms (Ag_236_), generated using an FCC-like metallic silver arrangement, with an Ag–Ag nearest-neighbor distance of approximately 2.89 Å and an approximate model diameter of 1.9 nm. This simplified cluster was used as a model-dependent representation of metallic silver and was not intended to reproduce the full-size experimental AgNP population. The constructed AgNP structure was positioned within an orthorhombic box and solvated with water molecules using the TIP3P water model (Jorgensen et al., 1983; Sirous et al., 2019; Brogi et al., 2020). The molecular dynamics calculations were performed using the OPLS force field (Jorgensen et al., 1996), with the aid of the CUDA API, using two NVIDIA graphics processing units (GPUs). To achieve a final salt concentration of 0.15 M, Na^+^ and Cl^−^ ions were added to simulate the physiological concentration of monovalent ions found in the body. The ensemble class NPT was used for molecular dynamics simulations, with a constant number of particles, pressure of 1.01325 bar, and temperature of 300 K. The RESPA integrator was used to calculate the equations with an inner time step of 2.0 fs, estimating the motion for bonded and non-bonded interactions within the short-range cutoff (Humphreys et al., 2002). The Nosé-Hoover thermostat technique was employed to maintain a constant temperature throughout the simulation (Hoover, 1985), with the Martyna-Tobias-Klein method being used to keep the pressure constant (Martyna et al., 1994). The van der Waals and short-range electrostatic interactions were kept constant at 9.0 Å, in contrast to long-range electrostatic interactions, which were calculated using the particle mesh Ewald technique (PME) (Essmann et al., 1995). The systems were progressively relaxed and brought to equilibrium using a standard procedure comprising several constrained minimizations and molecular dynamics simulations. The molecular dynamics simulations were performed in duplicate to improve the reliability of the results. The analysis tools within the Desmond package were employed to examine the molecular dynamics outputs generated during the simulation studies.

#### 2.7.2 Ligand preparation

A list of 19 phytochemical compounds of *S. bryopteris* that are well known and documented in the literature was selected. To investigate the mode of action of these phytocompounds against PARP-1, molecular docking investigation was conducted using AutoDock tools version 4.2 (Morris et al., 2009).

#### 2.7.3 Unveiling protein binding sites: using CASTp and UCSF chimera

The CASTp server was used to predict the potential binding sites of PARP-1 (http://sts.bioe.uic.edu/castp/) (Tian et al., 2018). The CASTp server displays the overall number of pockets as well as the amino acid residues involved in each pocket. The pockets were further arranged according to their area and volume. Using UCSF Chimera, we visualized the results of the CASTp server.

#### 2.7.4 Protein preparation

The RCSB Protein Data Bank (https://www.rcsb.org/) was used to obtain the structure of the PARP-1 protein (PDB ID 5DS3) in PDB format. Using the ‘Open Babel 2.4’ program, the 3D structures of the selected phytochemicals were retrieved from PubChem in SDF (structure data format) and converted to PDBQT (protein data bank, partial charge and atom type). Molecular docking was performed using AutoDock 4.2 and AutoDock Tools (Morris et al., 2009). The PDB structure of the protein was accessed using AutoDock Tools. Subsequently, water molecules, heteroatoms, and bound ligands were removed from the protein. Then, polar hydrogen and Kollman charges were added to the protein molecule. The grid parameter was chosen to encompass the active site of the protein. In this study, the grid spacing was set to 0.375 Å (as default) and center grid box was set to a *x* = 5.447, *y* = 36.952 and *z* = 10.174, respectively. The dimensions of x, y, and z were fixed at 80 × 80 × 90 grid points. The number of GA runs after the completion of the auto grid finished was thirty. The default values were applied to all other parameters.

#### 2.7.5 Molecular docking

Molecular docking was performed to understand how ligands interact with specific proteins. This is a useful and efficient technique for the identification of potential lead compounds in drug discovery. Virtual screening, in which small molecules are docked into known protein structures, is an important part of this process. Discovering novel candidate inhibitors for a specific target becomes feasible through virtual screening of small-molecule database. The Auto Dock tools make the process of finding potential new drug candidates faster and cheaper. Auto Dock 4.2 is a popular docking program, that uses a Lamarckian genetic algorithm to determine the interaction of ligands with the protein and their binding energy, which represents how ligands are bound to the target protein (Morris et al., 2009; Wadhwa et al., 2023a). Each docking software uses distinct search algorithms, which are techniques employed to anticipate the potential conformation of the binary complex.

#### 2.7.6 ADMET profiling

The SwissADME webserver was used to predict the pharmacokinetics and toxicity of the identified phytocompounds from the *S. bryopteris* extract (Daina et al., 2017). Drug-likeness studies evaluate a molecule’s bioavailability to predict its propensity to function as an oral medication. Understanding the various properties of ADMET is crucial in drug design, as undesirable properties of ADMET can lead to drug failure during clinical trials. Accordingly, this step provides accurate information regarding the drug-like characteristics of the phytochemicals present in *S. bryopteris*, including numerical values for parameters such as blood-brain barrier permeation, toxicity, and gastrointestinal absorption.

### 2.8 Anticancer activity of AgNPs

#### 2.8.1 Cell culture

Human ovarian carcinoma (SKOV3 and OVCAR-3) cells and normal human ovarian surface epithelial (IOSE80) cells were cultured separately in Minimum Essential Medium Eagle (MEM) supplemented with 10% fetal calf serum (FCS), 1% penicillin-streptomycin, and 2.2 g/L sodium bicarbonate. Cells were maintained in 100-mm culture dishes at 37 °C in a humidified incubator containing 5% CO_2_ and were subcultured at 80–90% confluence using a 1× trypsin-ethylenediaminetetraacetic acid (EDTA) solution.

#### 2.8.2 MTT assay

The MTT test was used to assess the cytotoxicity of AgNPs in different ovarian cell lines (Denizot and Lang, 1986). Cells were seeded into 96-well plates at approximately 5 × 10^3^ cells/mL. A 24 h incubation period was conducted at 37 °C with CO_2_ (5%). The next day, plant extract and AgNPs were added to the microtiter plates at concentrations ranging from 2 to 100 μg/mL for preliminary cytotoxicity screening. Based on the initial screening, selected concentrations around the cytotoxic range were further tested to determine the IC_50_ values. In particular, concentrations of 2.9, 5.8, 8.7, 11.6, and 14.5 μg/mL were tested in triplicate. After 24 h of incubation, 10 µL of freshly prepared MTT solution (5 mg/mL in 1× PBS, pH 7.4) was added to each well, and the cells were incubated for an additional 3 h. After 3 h, the formazan crystals were allowed to dissolve in 50 µL of isopropanol, and the absorbance was measured at 620 nm using a Spectro Star Nano Spectrophotometer (BMG Lab Tech., Germany) after 10 min. To minimize possible optical interference from AgNPs at the MTT reading wavelength, concentration-matched AgNP-treated wells without MTT reagent were included as background controls. The absorbance values obtained from these background wells were subtracted from the corresponding AgNP-treated wells before calculating cell viability/cytotoxicity. Wells without any drug treatment were considered as controls. The percentage cytotoxicity was calculated using formula (U − T)/U × 100, where U represents the mean optical density of untreated control cells and T represents the mean optical density of treated cells after subtraction of the corresponding concentration-matched AgNP background absorbance.

#### 2.8.3 Trypan blue exclusion assay

To further validate the MTT results and exclude possible misinterpretation due to AgNP-related optical interference, cell viability was also evaluated using the Trypan Blue exclusion assay. The cell lines were grown on 35 mm cell culture plates for overnight at 37 °C, 5% CO_2_ incubator. Next day, cells were treated with IC_50_ doses of plant extract and AgNPs for 24 h. After incubation, cells were scrapped and centrifuged at 2000 rpm for 10 min. Pellets were resuspended in 1 × PBS (pH 7.4) and trypan blue dye (0.04%) was added at 1:1 ratio. The 10 µL of that mixture was loaded on haemocytometer: visualized under brightfield microscope (Magnus MLXi) and viable, non-viable cells were quantified.

#### 2.8.4 Detection of intracellular reactive oxygen species (ROS)

Cancer cells were grown on cover slips in 35 mm petri plates in a CO_2_ incubator at 37 °C for 24 h. After 24 h, cells were treated with IC_50_ values of AgNPs and plant extracts for 24 h. After incubation, cells were treated with H_2_O_2_ (0.03%) for 30 min (positive control) along with the negative control. Cells were washed twice with PBS (pH 7.4) and incubated with 2 µg/mL 2′,7′-dichlorodihydrofluorescein diacetate (H_2_DCFDA), prepared in cell-culture-grade methanol, for 30 min at 37 °C in a humidified atmosphere containing 5% CO_2_ and protected from light. Following incubation, the cells were immediately washed twice with PBS to remove extracellular H_2_DCFDA. The *S. bryopteris* leaf extract was included as an additional treatment control to evaluate whether extract-derived phytochemicals contributed to ROS production independently of the AgNP formulation. The slides were prepared by inverting the coverslips having cells on cleaned slides on 20% glycerol drop and observed under ×40 magnification by fluorescence microscope, Olympus, excitation at 480 nm and emission at 526 nm. The fluorescence intensity data were plotted to generate the intensity graph.

## 3 Results

### 3.1 GC-MS analysis of phytocompounds present in leaf extract of *Selaginella bryopteris*

**FIGURE 3:**
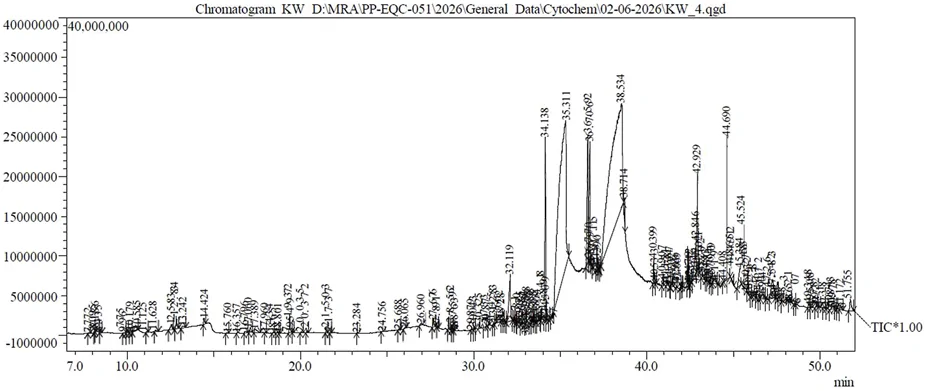
GC-MS chromatogram of leaf extract of *Selaginella bryopteris*.

GC-MS analysis suggested that the leaf extract of *S. bryopteris* contains several phytocompounds, including fatty acids, α-tocopherol, γ-tocopherol, β-sitosterol, γ-sitosterone, thunbergol, eicosanoic acid, catechol, furaneol, azelaic acid, behenic alcohol, heneicosane, and other bioactive constituents. These compounds may contribute to the reduction, capping, and stabilization of AgNPs, as reported in Supplementary Table S1, with their corresponding retention times shown in Figure 3.

### 3.2 ICP-MS analysis of leaf extract of *Selaginella bryopteris*

ICP-MS analysis was performed to identify and quantify the inorganic components and micronutrients present in the leaf extract of *S. bryopteris* (Table 1). This analysis was used only to determine the elemental composition of the extract, whereas the phytochemical constituents were investigated by GC-MS analysis and are reported in Supplementary Table S1. Trace elements play an important role in plant metabolism and in the formation of phytochemical constituents. Some metals, such as zinc, iron, chromium, copper, and cobalt, are generally tolerated at low concentrations but may become toxic when present at higher levels. In contrast, other metals, including mercury, cadmium, and lead, are toxic even at low concentrations. Therefore, determining the elemental composition of plant extracts is essential to assess their safety and support their potential use in medicinal applications (Table 1).

**TABLE 1:** Elemental composition of the dried leaf extract of *Selaginella bryopteris*, with concentrations expressed in ppb (parts per billion).

Name of element	Mass	Concentration	Units
Na	23	63.483	ppb
Zn	66	<0.000	ppb
Se	77	11.099	ppb
Se	78	7.709	ppb
Se	82	6.817	ppb
Li	7	7.298	ppb
Na	23	53.224	ppb
Mg	24	48.893	ppb
Al	27	18.775	ppb
K	39	96.593	ppb
Ca	43	19.175	ppb
Ca	44	64.880	ppb
Cr	52	0.700	ppb
Mn	55	2.956	ppb
Fe	56	18.597	ppb
Co	59	0.592	ppb
Ni	60	1.215	ppb
Cu	63	0.787	ppb
Zn	66	<0.000	ppb
Se	78	5.954	ppb
Sr	88	1.134	ppb
Te	125	30.593	ppb
Ba	137	0.922	ppb
Tl	205	28.201	ppb
[Pb]	206	3.131	ppb
[Pb]	207	3.065	ppb
Pb	208	3.068	ppb
Bi	209	4.455	ppb

### 3.3 Green synthesis of AgNPs and their characterization

#### 3.3.1 Biosynthesis of AgNPs

**FIGURE 4:**
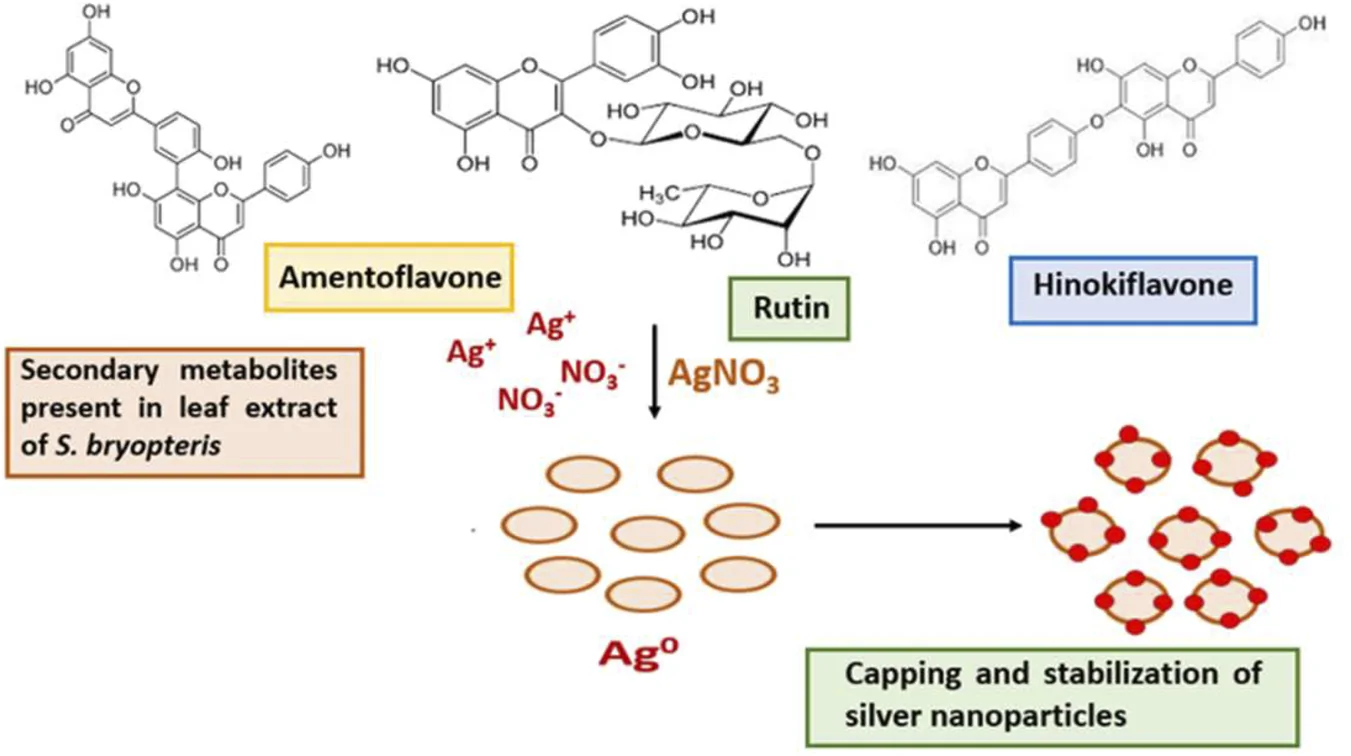
Schematic representation of mechanism of plant mediated biosynthesis of AgNPs using aqueous leaf extract of *Selaginella bryopteris.*

A significant color change in the solution preliminarily confirms the green synthesis of AgNPs. The color changes from pale yellow to dark red brown, indicating the reduction of silver ions in the aqueous solution by the capping agent present in the leaf extract of *S*. *bryopteris* (Figure 4). Although the synthesis of *S. bryopteris*-derived AgNPs was performed according to our previously reported protocol, the essential physicochemical characterization data relevant to the AgNPs evaluated in the present biological study are reported here to provide a self-contained description of the NPs system.

#### 3.3.2 Characterization of the green synthesized AgNPs

##### 3.3.2.1 UV–visible absorption spectroscopy

**FIGURE 5:**
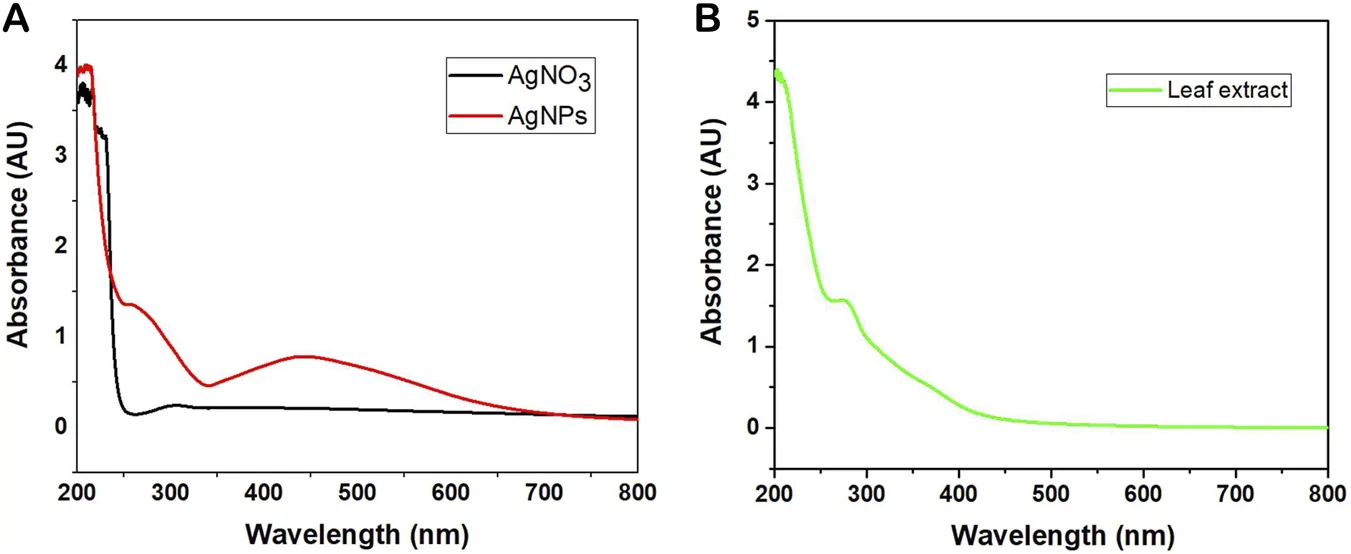
UV–visible absorption spectra of **(A)** 1 mM AgNO_3_ solution and green-synthesized AgNPs and **(B)** appropriately diluted *Selaginella bryopteris* leaf extract. The diluted leaf extract showed a resolved absorption feature with a maximum at approximately 275 nm, whereas the AgNP suspension exhibited the characteristic surface plasmon resonance band at approximately 425 nm.

This method revealed a broad absorption band in the visible region, which can be attributed to the color change associated with the excitation of free electrons and the formation of the SPR band. This phenomenon occurs because the conduction and valence bands of metallic NPs are close to each other. The characteristic SPR band observed at 425 nm suggests the complete reduction of silver ions and the formation of AgNPs (Figure 5A). To avoid signal saturation in the UV region, the *S. bryopteris* leaf extract was diluted 11-fold before spectral acquisition. The diluted extract displayed a distinct absorption feature with a maximum at approximately 275 nm (Figure 5B). This band may be associated with extract-derived phytoconstituents, including phenolic compounds, flavonoids, and other aromatic metabolites. These phytocompounds mainly undergo π–π* and n–π* transitions, resulting in characteristic absorption in UV region.

##### 3.3.2.2 Shape and morphology of green-synthesized AgNPs

**FIGURE 6:**
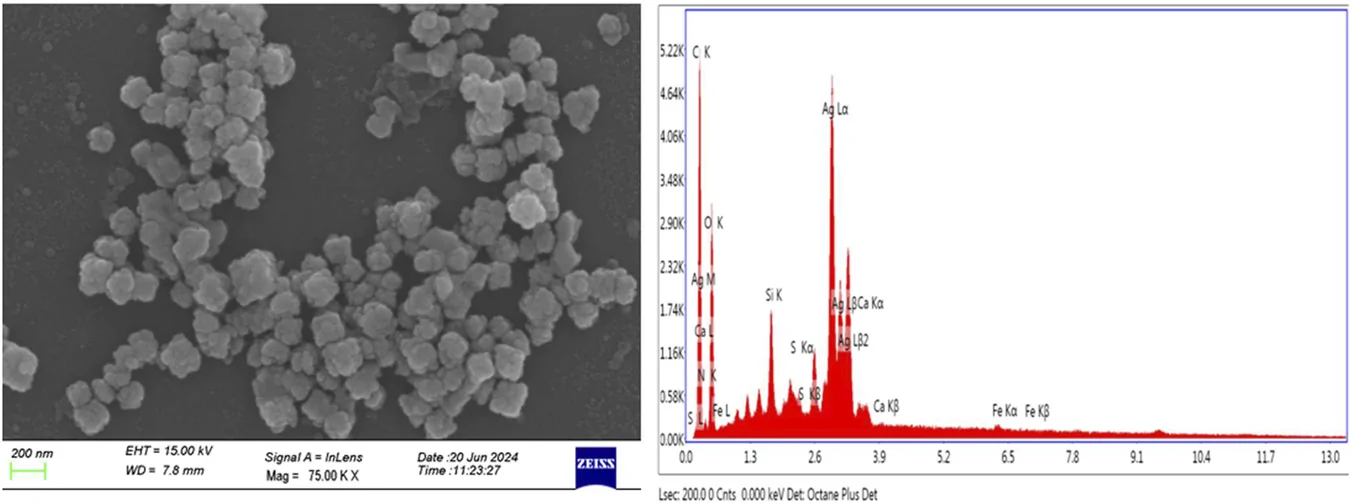
FESEM and EDX mapping of green synthesized AgNPs using aqueous leaf extract of *Selaginella bryopteris* (Image taken at magnification, 75 KX, scale bar 200 nm).

The shape and morphology of the green-synthesized AgNPs were investigated by FESEM analysis, which revealed that the NPs exhibited a predominantly spherical morphology. The elemental composition of the AgNPs was further confirmed by energy-dispersive X-ray spectroscopy (EDX), showing a strong signal at approximately 3 keV, corresponding to the characteristic silver peak, as shown in Figure 6.

**FIGURE 7:**
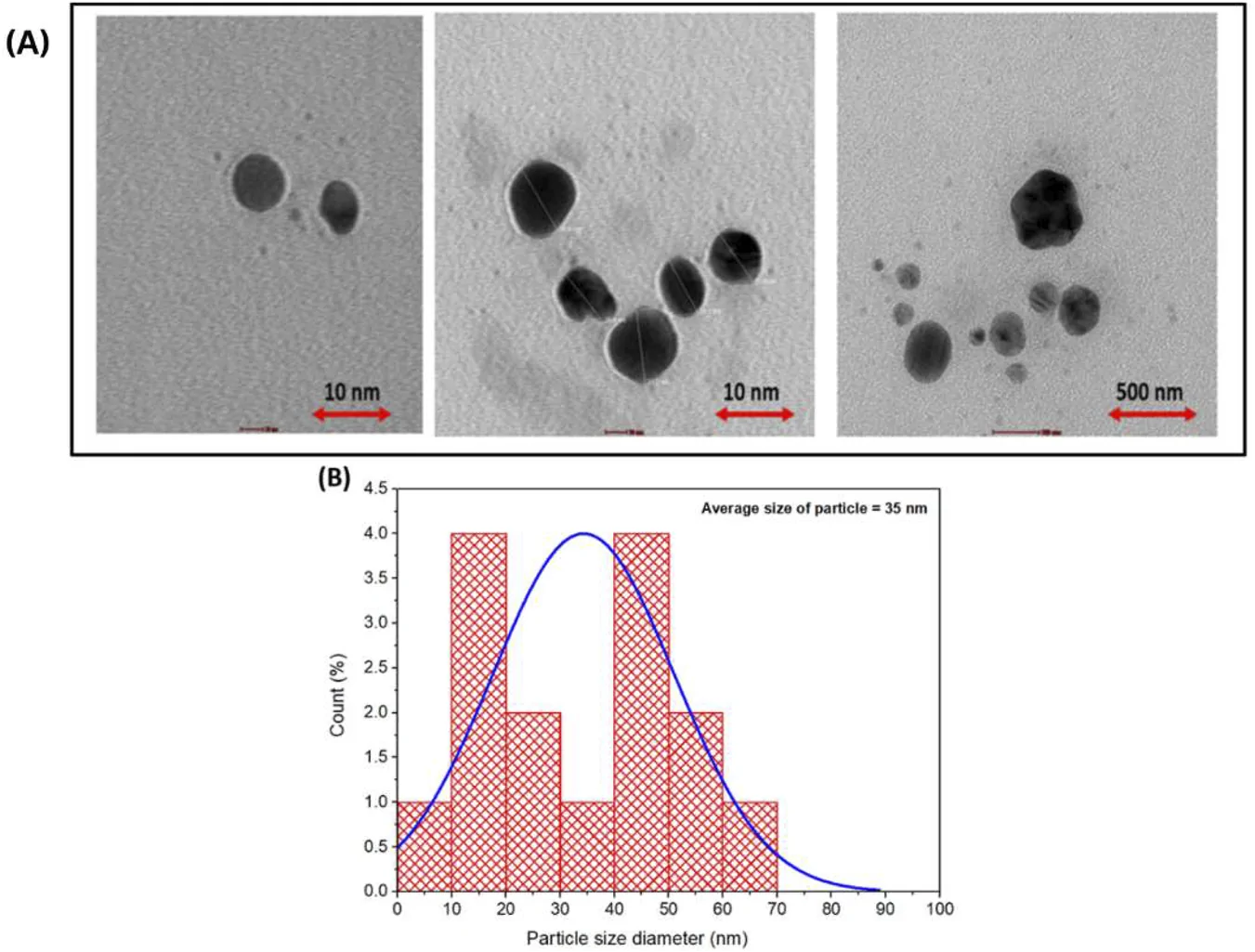
**(A)** TEM images of green-synthesized AgNPs at two different scales, 10 nm and 500 nm. **(B)** Particle size distribution histogram of the AgNPs.

TEM analysis further confirmed the size, shape, and morphology of the AgNPs (Figure 7A), revealing a predominantly spherical morphology with average size of 35 nm, as shown in Figure 7B.

##### 3.3.2.3 FTIR analysis

**FIGURE 8:**
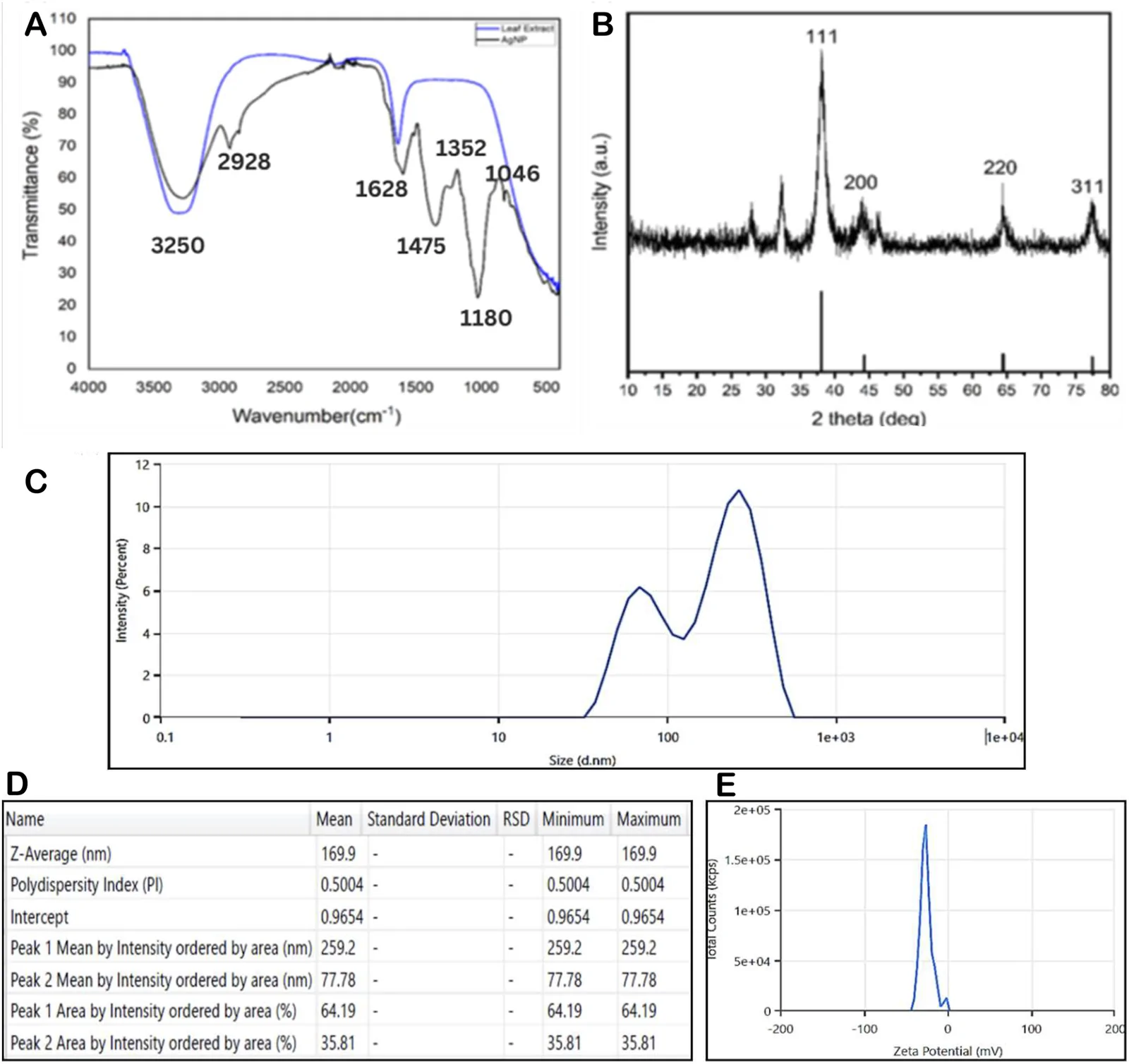
**(A)** FTIR spectra and **(B)** XRD pattern of green synthesized AgNPs using leaf extract of *Selaginella bryopteris*. **(C and D)** DLS analysis and **(E)** zeta potential of green synthesized AgNPs.

In one of our previous studies (Yadav et al., 2020), *S. bryopteris*-derived AgNPs were characterized using FTIR and X-ray diffraction (XRD) techniques (Figure 8). FTIR analysis was performed to identify the functional groups of phytoconstituents potentially involved in the reduction, capping, and stabilization of AgNPs (Figure 8A). AgNPs exhibited a distinct spectral peak at 3,250 cm^−1^ showing the symmetric vibration of O-H, while the peak observed at 2,928 cm^−1^ showed asymmetric vibration of aliphatic C-H bonds. The peak observed at 1,628 cm^−1^ indicates the presence of a C=O bond, the peak at 1,475 cm^−1^ is categorized as C=C, the peak obtained at 1,356 cm^−1^ is related to C-N bond. In addition, the peaks obtained at 1,180 cm^−1^ indicated the presence of O-H groups of phenols (Mughal et al., 2024; Tahir et al., 2024; Tanveer et al., 2024). The observed peak at 1,046 cm^−1^ indicates a C-C stretch, C-N aromatic amino acid, or a C=C bond of an aromatic ring. The bands at 1,356 cm^−1^ indicate C-C and C-N stretches whereas at 1,628 cm^−1^ they indicate the stretching vibrations of the primary amines. Bands at 3,156, 3,236, 3,300 and 3,376 cm^−1^ indicate the presence of phenol and alcohol groups; a peak at 3,484 cm^−1^ indicate O-H stretching vibrations of a phenol group (Figure 8A). Accordingly, the FTIR spectrum of the green-synthesized AgNPs showed characteristic absorption peaks corresponding to functional groups potentially involved in NPs formation and stabilization. These findings suggest that phytoconstituents from the *S. bryopteris* leaf extract may contribute to Ag^+^ reduction, capping, and stabilization of the synthesized AgNPs. XRD analysis showed characteristic diffraction peaks for AgNPs at approximately 2θ values of 38.1°, 44.0°, 64.5°, and 77.4°, which were assigned to the (111), (200), (220), and (311) crystallographic planes, respectively. The 2θ values and corresponding Miller indices (hkl), shown in Figure 8B, are consistent with the face-centered cubic (FCC) structure of metallic silver, according to JCPDS file no. 04-0783. The additional diffraction peaks observed may be attributed to the presence of organic phytocompounds on the surface of AgNPs (Figure 8B).

The hydrodynamic diameter of the green-synthesized AgNPs was 169.9 nm, with a polydispersity index (PDI) of 0.5. This value was larger than the particle size observed by TEM, as DLS measures NPs in the aqueous state, whereas TEM evaluates them in the dry state. Moreover, DLS also accounts for the hydrodynamic shell surrounding the NPs, including capping and stabilizing agents attached to the AgNP surface (Figures 8C,D). The zeta potential of the green-synthesized AgNPs was −25.39 mV, indicating good colloidal stability of the NPs (Figure 8E). DLS analysis was performed to determine the hydrodynamic size distribution of the green-synthesized AgNPs, whereas zeta potential analysis was used to assess their surface charge and colloidal stability. Together with UV–visible spectroscopy, electron microscopy, EDX, XRD, FTIR, and thermal analysis, these results support the successful formation and physicochemical characterization of the AgNP system.

##### 3.3.2.4 TGA and DTG analyses of the green-synthesized AgNPs

**FIGURE 9:**
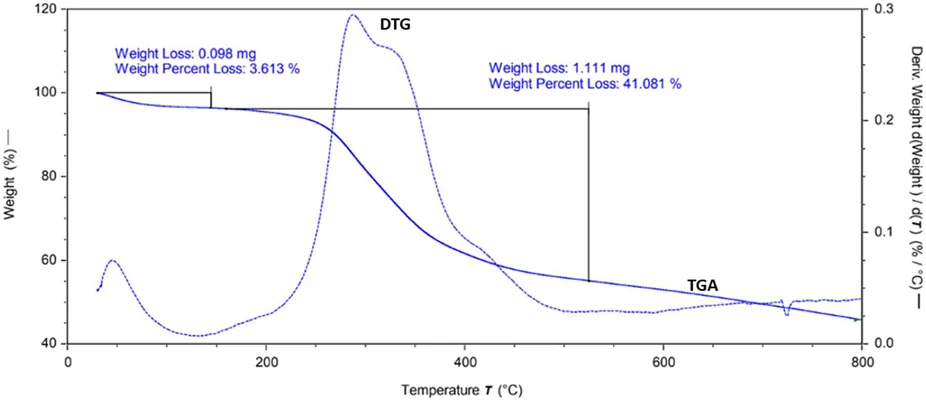
TGA and DTG thermal profiles of green-synthesized AgNPs.

The AgNP sample was analyzed over a temperature range of 23 °C–800 °C. The TGA curve showed a slight weight loss of approximately 3.613% between 23 °C and 250 °C, mainly attributable to the loss of physically adsorbed water molecules or surface moisture from the AgNPs. A major weight loss of 41.081% was observed in the temperature range of 250 °C–530 °C, corresponding to the decomposition of organic phytocompounds, such as polyphenols, tocopherols, and flavonoids, adsorbed on the AgNP surface as capping agents. Only a minor weight loss was observed between 530 °C and 800 °C, after which the sample became thermally stable, consistent with the thermal stability of metallic silver in this temperature range. The residual mass at 800 °C was approximately 55.306%. Thus, the AgNPs formulation was composed of approximately 3.6% adsorbed water/surface moisture, 41.1% extract-derived organic material, and 55.3% inorganic residue, mainly corresponding to metallic silver. These findings indicate that the green-synthesized AgNPs obtained from the leaf extract of *S. bryopteris* contain approximately 55% silver by weight and confirm the presence of a phytochemical capping layer around the AgNPs (Figure 9).

##### 3.3.2.5 ICP-MS

The silver content of the green-synthesized AgNPs was determined by ICP-MS using a calibration curve prepared with standard solutions ranging from 10 to 200 ppb. The reagent blanks showed negligible silver concentrations, ranging from 0 to 0.589 ppb. After correcting the respective dilution factors, the silver concentration in the AgNP samples was found to be 16.934 and 17.975 ppm at 1000-fold and 100-fold dilutions, respectively. The close agreement between the concentrations obtained using two different dilution factors supports the accuracy and reproducibility of the ICP-MS measurements. Together with the TGA/DTG results, the ICP-MS data provide a quantitative estimate of the AgNP formulation composition and support the definition of the actual silver content administered in the biological assays.

##### 3.3.2.6 pH and thermal stability of green synthesized AgNPs

**FIGURE 10:**
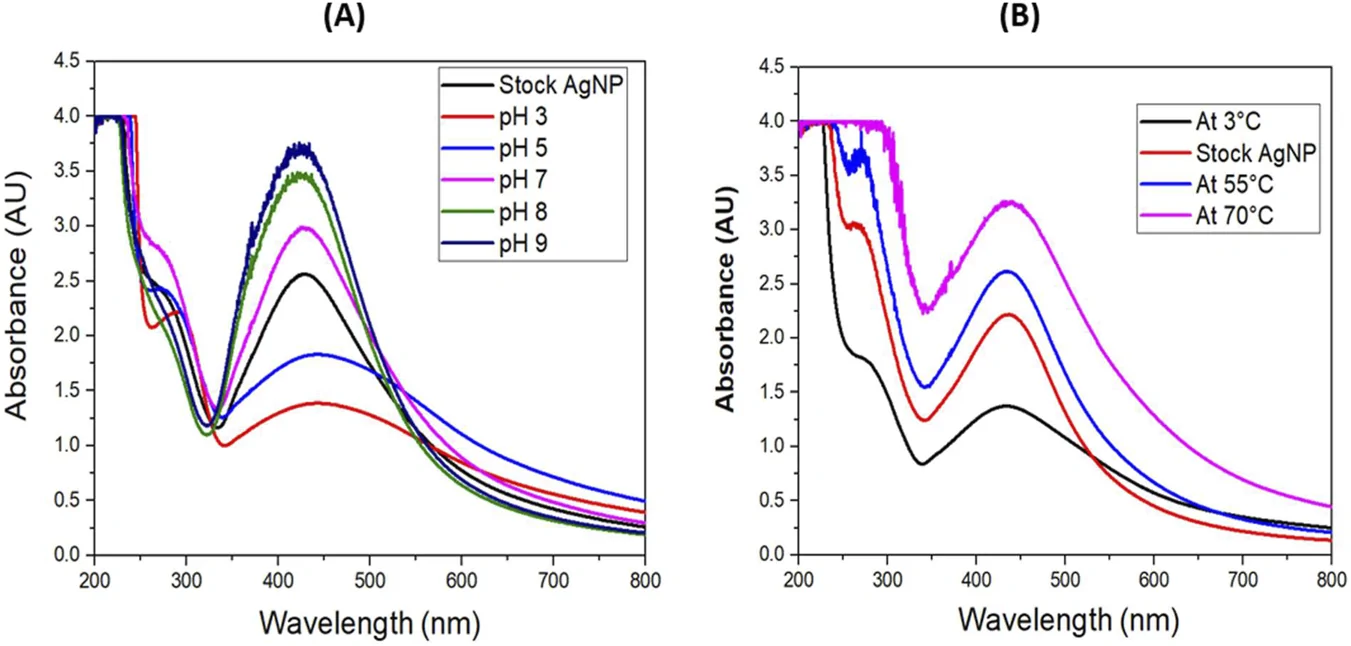
UV–visible spectra of green-synthesized AgNPs recorded to assess their stability at different pH values **(A)** and temperatures **(B)**.

The pH value is a crucial factor that facilitates the stabilization and coating ability of phytocompounds. It plays a crucial role in determining the particle size of NPs as it can modify the functional groups of phytocompounds, which further alters their reducing and capping ability (Akhtar et al., 2024a). The pure stock AgNPs exhibited a clean and narrow SPR with a peak absorbance of 2.5 at 423 nm. The addition of 10% HNO_3_ lowered the peak absorbance to 1.75 and 1.25, respectively, with a very broadened SPR. The peak obtained for pH solutions 3 and 5 indicated a broad peak of absorption spectra indicating non-uniform (heterogenous) AgNPs. At pH (3 and 5), the SPR broadens by indicating the formation of larger sized particles that were formed due to increased particle aggregation. At pH 7, 8, and 9, the maximum formation of AgNPs was observed compared to the stock with a peak absorbance of 3.0, 3.4, and 3.75, SPR at 423 nm. According to the UV-visible spectroscopy results, alkaline pH conditions had a more favorable effect on AgNP stability than acidic conditions. AgNPs exhibit higher absorbance at higher pH values (Figure 10A) (Akhtar et al., 2024a; Subhani et al., 2024). The effect of temperature on AgNP stability was evaluated at 3, 55, and 70 °C, as well as at room temperature. The maximum SPR absorption peak was observed at 70 °C, likely due to increased molecular motion and enhanced reduction of silver ions, which may promote NPs formation. The increase in AgNP absorbance may therefore reflect an increased rate of NPs synthesis (Figure 10B) (Akhtar et al., 2024b).

### 3.4 MTT assay

**FIGURE 11:**
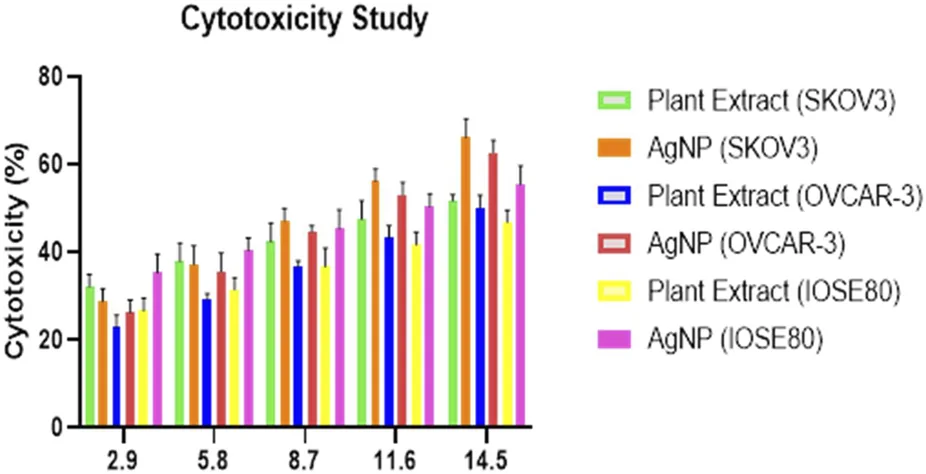
Cytotoxicity percentage of AgNPs at different concentrations, evaluated using the MTT assay.

**FIGURE 12:**
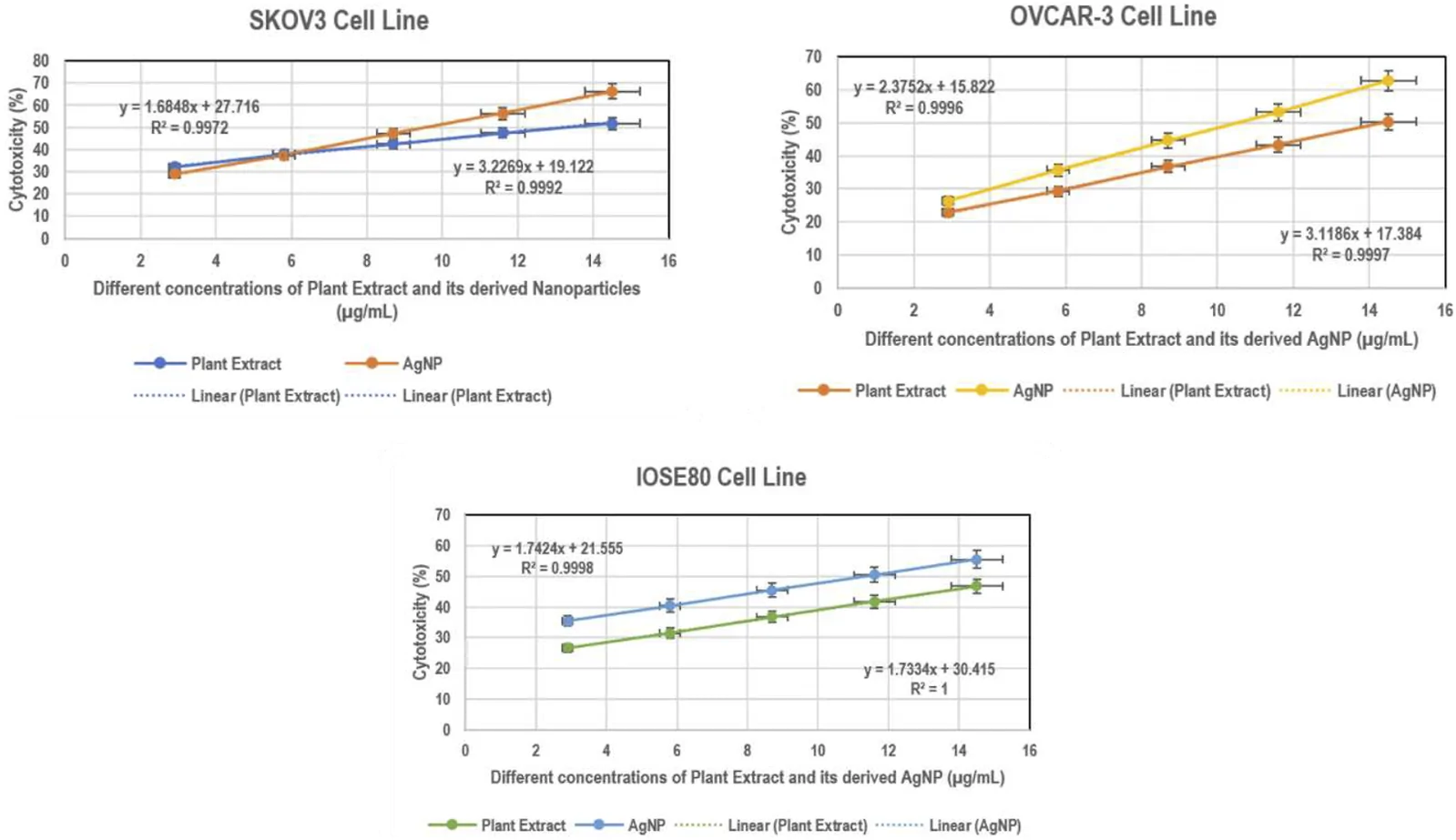
Determination of the IC_50_ values of AgNPs and *Selaginella bryopteris* leaf extract against SKOV3, OVCAR-3, and IOSE80 cell lines.

**FIGURE 13:**
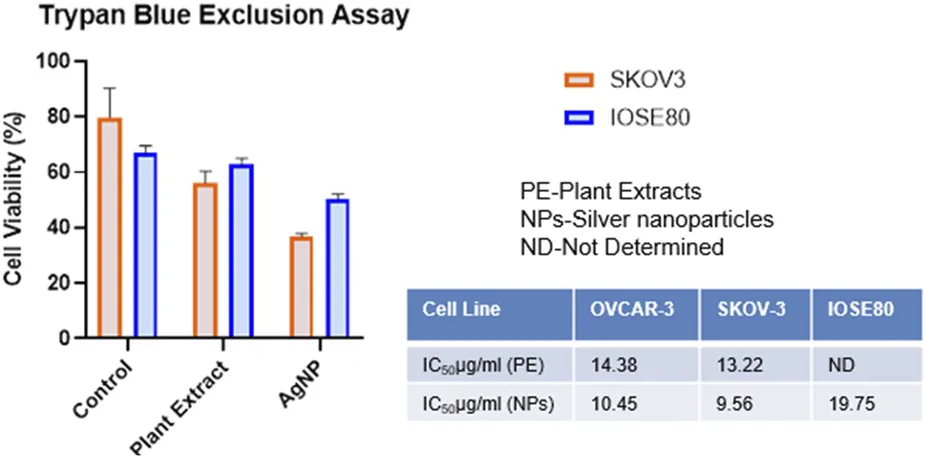
Cell viability studies performed using the Trypan blue exclusion assay.

The MTT assay was used to evaluate the viability of cells receiving pharmacological treatments. Cytotoxic medications target and prevent cell division by interfering with normal physiological processes. Our results indicate that AgNPs, which are concentration-dependent and decrease cell viability as the drug concentration increases, are the primary source of toxicity. In parallel, the non-tumorigenic ovarian epithelial cell line IOSE80 was included to preliminarily assess tumor-selective cytotoxicity. AgNPs showed lower cytotoxicity toward IOSE80 cells than toward SKOV3 and OVCAR-3 ovarian cancer cells, suggesting a more pronounced effect on cancer cells under the tested conditions. However, further studies are required to fully define the therapeutic window and tumor selectivity of the AgNP formulation. Significant cytotoxicity was observed starting from a concentration of 9 μg/mL. The drug concentration required to reduce cell viability to its initial 50% (IC_50_) was calculated from the equation reported in the cytotoxicity graph (Figures 11, 12). The Trypan Blue exclusion assay further confirmed that AgNPs decreased cell viability in both SKOV3 and OVCAR-3 cell lines (Figure 13).

**FIGURE 14:**
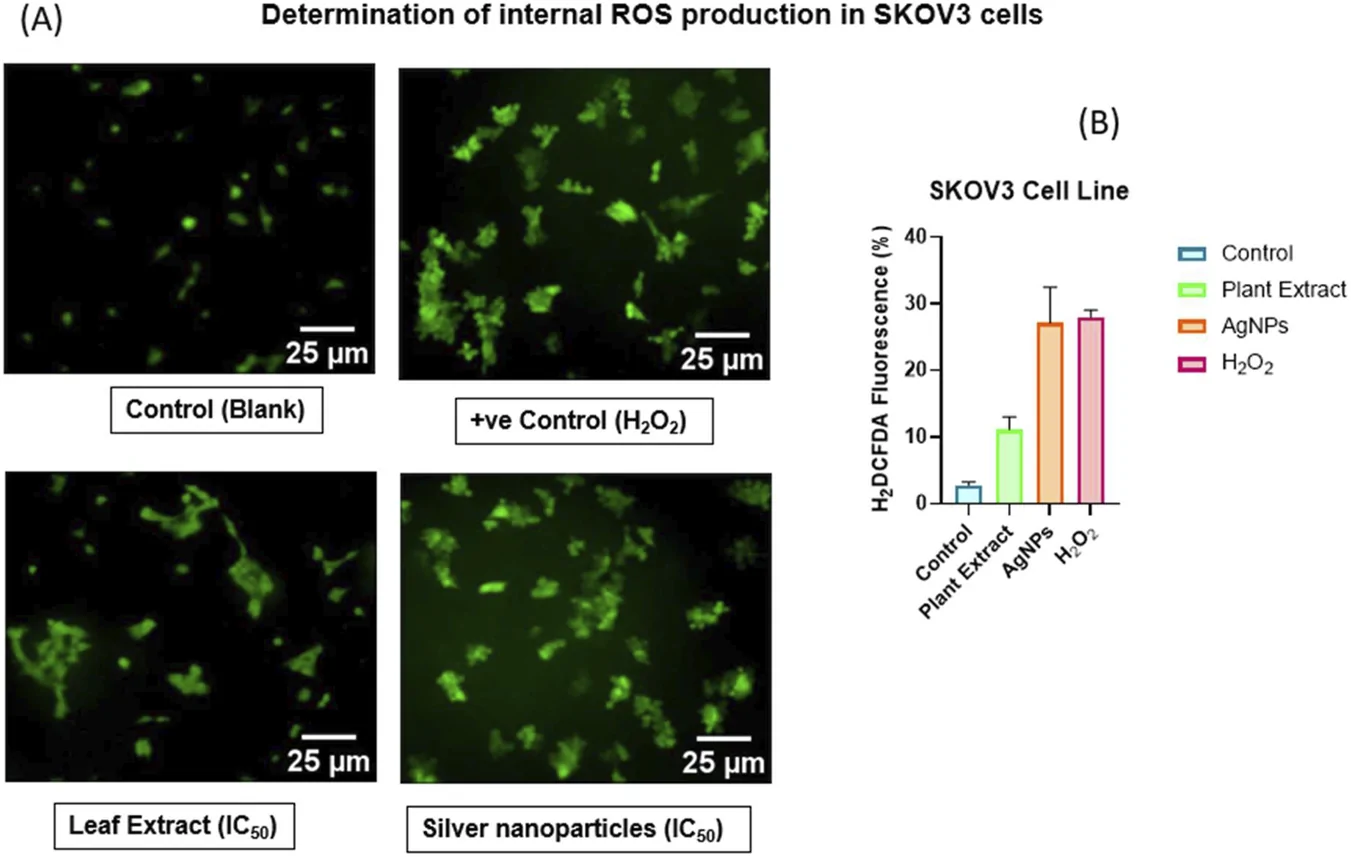
Determination of ROS production using 2′,7′-dichlorodihydrofluorescein diacetate (H_2_DCFDA). **(A)** Representative fluorescence microscopy images of SKOV3 cells acquired at ×40 magnification. Untreated cells maintained in growth medium were used as the blank control, while cells treated with 0.03% hydrogen peroxide were used as the positive control. Cells were treated with AgNPs or *Selaginella bryopteris* leaf extract at the corresponding IC_50_ concentrations. **(B)** Quantification of fluorescence intensity obtained from the corresponding images.

This discovery implies that AgNPs, whose activities are concentration-dependent, reduce cancer cell viability. Reactive oxidizing molecules, which can be radical and non-radical (H_2_O_2_), have hazardous effects on living cells. They can be produced exogenously or endogenously. Similar to cellular biomolecules, these molecules can easily remove electrons from (oxidize) molecules with which they remain in contact. This initial reaction can generate chain reactions that lead to apoptosis. Internal ROS production by 2՛,7՛-dichloro dihydrofluorescein diacetate (H_2_DCFA) (non-fluorescein in reduced form) as an indicator of ROS in cells was determined. The nonfluorescent H_2_DCFDA was converted to significantly fluorescent 2՛,7՛-dichlorofluorescein (DCF) upon cleavage of acetate groups by intracellular esterases and oxidation, which could be detected by fluorescence intensity. Very minute fluorescence was observed in the control and ROS-induced fluorescence in the positive control. ROS production was markedly increased in AgNP-treated cells compared with untreated control cells. The *S. bryopteris* leaf extract was also evaluated as an additional control and showed a lower ROS-associated fluorescence signal than AgNPs under the tested conditions. These findings suggest that the ROS response is mainly associated with the AgNP formulation, although a possible contribution of extract-derived phytochemicals bound to the AgNP surface cannot be completely excluded (Figure 14).

Reactive oxidizing molecules derived from ROS have an impact on cell survival as they are endogenously produced (Li et al., 2015). They are defined as reactive molecules or ions formed by the incomplete one-electron reduction of oxygen. Superoxide (∙O_2_^−^), hydrogen peroxide (H_2_O_2_), and hydroxyl radicals (∙OH)are the main types of ROS produced in the cell. The maximum concentration of ROS in cells is thought to be produced by the mitochondria (Collins et al., 2012).

### 3.5 Computational studies on AgNPs

#### 3.5.1 Exploratory molecular dynamics analysis of a simplified silver cluster model

**FIGURE 15:**
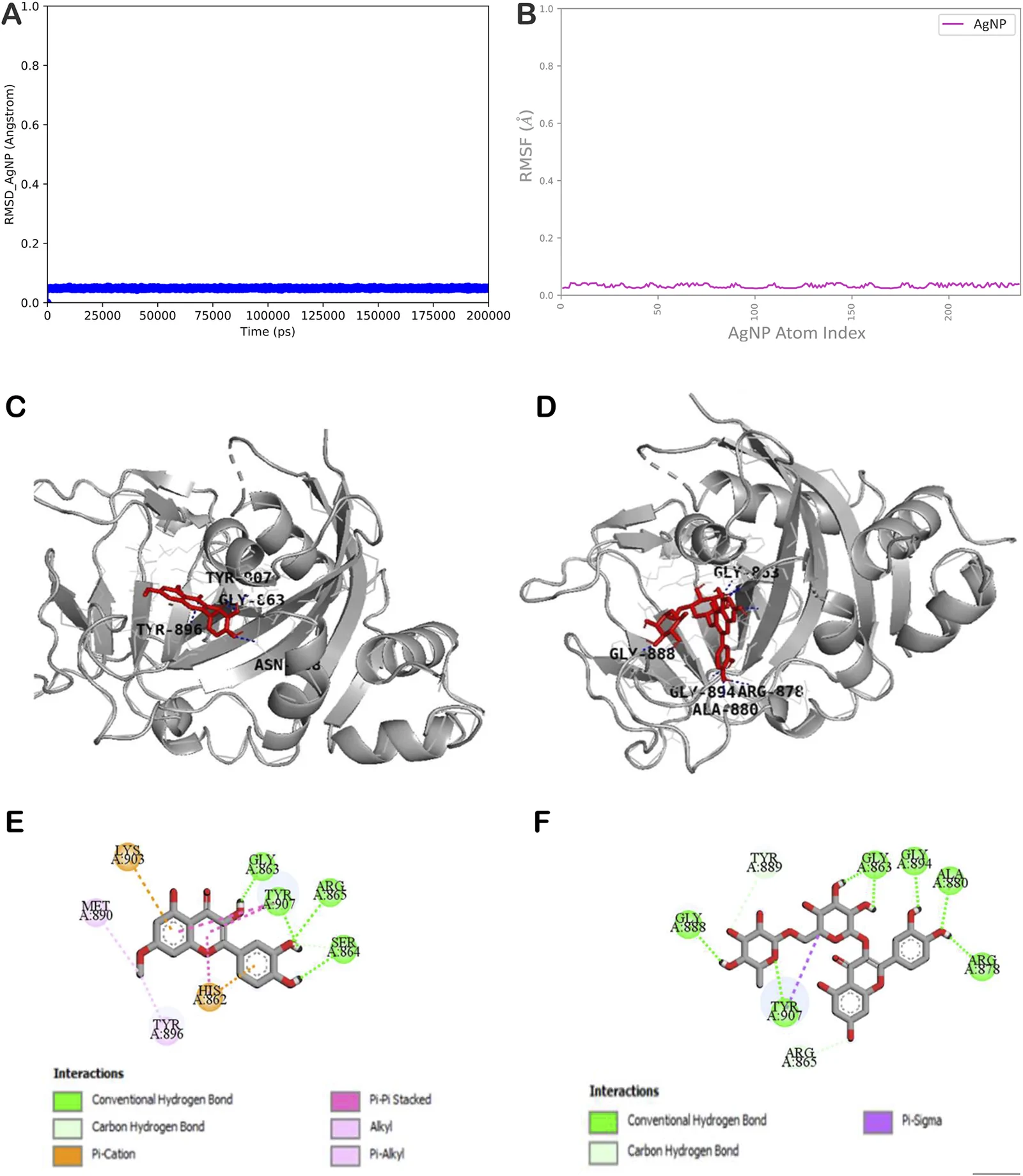
**(A)** RMSD and **(B)** RMSF analysis of the AgNP. 3D interaction established by **(C)** rhamnetin and **(D)** rutin against PARP-1, 2D interaction of **(E)** rhamnetin and **(F)** rutin with PARP-1.

Molecular dynamics simulation has evolved into a fundamental *in silico* technique for capturing the dynamic events of biological systems under specific physiological environment conditions. A high-performance molecular dynamic can provide detailed insight into the structural changes and internal interactions of selected complexes on the time scales evaluating atomic-level perturbations. Accordingly, molecular dynamics simulation experiments were performed to investigate the behavior and stability of the AgNP. For the molecular dynamics analysis, the AgNP system was modeled as an atomistic silver cluster (Ag_236_). This model was used as a simplified nanoscale representation of metallic silver to explore the structural behavior of the cluster under the applied simulation conditions. The overall structural fluctuations of the Ag_236_ cluster model were evaluated by RMSD and RMSF analysis (Figures 15A,B). Under the applied simulation conditions, the modeled cluster showed limited structural fluctuations, suggesting that the simplified atomistic silver cluster remained stable during the simulation. However, because metallic nanoparticles are complex non-conventional systems and the experimentally synthesized AgNPs represent a heterogeneous colloidal population, these simulations should be interpreted as exploratory and model-dependent. Therefore, the molecular dynamics analysis was used only as a complementary computational observation and not as definitive evidence of the stability or biological behavior of the experimentally obtained AgNP formulation. Accordingly, the experimental characterization data, including TEM, DLS, zeta potential, XRD, TGA, and ICP-MS, remain the primary evidence supporting AgNP formation, stability, crystallinity, and composition.

#### 3.5.2 Molecular docking and ADMET profiling

The phytocompounds selected for the computational analysis included compounds identified in the present GC-MS analysis of *S. bryopteris* leaf extract, together with flavonoids and biflavonoids previously reported in *S. bryopteris* and related *Selaginella* species. It should be noted that not all docked phytocompounds were experimentally identified in the present extract. In particular, high-molecular-weight and polar flavonoids/biflavonoids may not be efficiently detected by standard GC-MS analysis because of their limited volatility and thermal stability. Therefore, the docking analysis should be considered an exploratory, literature-supported screening, and future LC-MS/MS or HPLC-based phytochemical profiling will be required to confirm the presence and abundance of these compounds in the studied extract.

##### 3.5.2.1 Binding site prediction

The active binding site of the PARP-1 (PDB ID 5DS3) was predicted using the CASTp server. A total of 31 active pockets were identified, with the largest pocket having an area of 317.23 Å^2^ and volume of 385.35 Å^3^. The UCSF chimera tool was used to eliminate cavities with zero openings, resulting in the identification of only 12 cavities out of 31 active pockets. Supplementary Figure S1 shows the amino acid residues in the active pocket.

##### 3.5.2.2 Interaction analysis

The role of different phytochemicals present in *S. bryopteris* was analyzed to identify the most promising PARP-1 inhibitors. The goal of molecular docking is to determine the binding mode of ligand to a protein using a search algorithm approach. To achieve an optimized arrangement of both interacting molecules to reduce the overall free energy of the system. The final predicted binding free energy (ΔG_bind_) is expressed through various factors, such as dispersion and repulsion (ΔG_vdw_), hydrogen bonding (ΔG_hbond_), desolvation (ΔG_desolv_), electrostatic interactions (ΔG_elec_), torsional free energy (ΔG_tor_), the ultimate total internal energy (ΔG_total_), and the energy of the unbound system (ΔG_unb_). Therefore, gaining a comprehensive understanding of the fundamental principles governing the predicted binding free energy (ΔG_bind_) offers valuable insights into the nature of the various interactions that facilitate the docking of molecules (Meng et al., 2011). Based on the Lamarckian Genetic Algorithm, AutoDock 4.2 facilitates the analysis of protein-ligand interactions by identifying the lowest binding energy score, indicating that the ligand has formed several interactions with the enzyme. The interaction between selected phytochemicals and PARP-1, their binding energies and structure of best hit flavonoids compounds interacted with target PARP-1, including their binding energies and the binding poses of the best-hit flavonoid compounds within the PARP-1 target protein, are shown in Supplementary Figure S2. These parameters were carefully analyzed to identify potential PARP-1 inhibitors (Supplementary Table S2). In addition, the ADMET profiles of drug-like compounds with significant binding energies were also calculated (Supplementary Table S3). It should be noted that these ADMET predictions refer only to the selected phytochemicals identified in *S. bryopteris* extract and not to the green-synthesized AgNP system as a whole. This analysis suggested that rhamnetin and rutin are among the most promising *S. bryopteris* phytoconstituents showing favorable predicted interactions with PARP-1. The molecular docking output is shown in Figures 15C–F. Rutin exhibited favorable binding interactions with this receptor, exhibiting a binding energy of −16.13 kcal/mol (Supplementary Table S2). Notably, it forms a π-sigma bond with Tyr907, and a hydrogen bond was established with Gly863 within the selected active binding site (Figures 15D,F). Rhamnetin showed a docking score of −11.56 kcal/mol (Supplementary Table S2) and interacted with His862, Gly863, Ser864, Arg865, Tyr896, Lys903, and Tyr907 from the selected active binding site (Figures 15C,E). Rhamnetin, a flavonoid phytocompound present in *S. bryopteris* extract, targets the ADP and NAD^+^ binding sites at the PARP-1 catalytic domain and interacts with key residues of the protein, including Gly863, Tyr907, Met890, and Lys903 (Figures 15C,E). Notably, the residues targeted by the selected natural products are well recognized as relevant for inhibiting PARP-1 (Shridhar Deshpande et al., 2021). As mentioned, the drug-likeness of the phytocompounds was evaluated by considering various ADMET-related characteristics, including pharmacokinetics, physiological parameters, water solubility, lipophilicity, *etc.* (Supplementary Table S3). Remarkably, among the various lead candidates, rhamnetin showed the most promising potential because of the absence of Lipinski’s rule of five violations according to the SwissADME calculation. The number of rotatable bonds for rhamnetin was less than 10, with a hydrogen bond acceptor function under 10 and a hydrogen bond donor function under 5. The molecular weight of rhamnetin was less than 500 kDa. Supplementary Figure S3 summarizes the drug likeness of rhamnetin by the bioavailability radar plot. The light pink region denotes the ideal range for each of the following characteristics: solubility, logS not greater than 6; saturation, fraction of carbons in the sp^3^ hybridization not less than 0.25; flexibility, no more than 9 rotatable bonds; TPSA, topological polar surface area less than 150 Å^2^. In addition, the binding of silver species, including Ag, Ag^+^, and Ag_3_, was investigated within the active site of the PARP-1 target protein. These silver species were found to interact with several PARP-1 amino acid residues, including Arg847, Leu854, His855, Asn856, Glu884, Asp899, Met900, Asn987, Ala995, Val997, and Gly927, as shown in Supplementary Figure S4.

## 4 Discussion

The use of plant-derived compounds for cancer treatment has been extensively investigated. Flavonoids, the secondary metabolites responsible for the color and flavors of many vegetables, have already been recognized for their therapeutic potential against several pathological conditions, including cancer. Due to the limited effectiveness and relevant adverse effects of current anticancer therapies, there is a growing interest in identifying new, effective, and safer agents, particularly compounds able to modulate multiple biological targets while minimizing undesired effects. Although the green synthesis of AgNPs has been widely investigated, the main novelty of this study lies in the integrated evaluation of *S. bryopteris*-derived AgNPs as potential anticancer agents against ovarian cancer cells, combining physicochemical characterization, cytotoxicity assays, ROS analysis, and PARP-1-focused *in silico* investigation of plant-derived phytoconstituents.

In the present study, *in vitro* experiments showed that AgNPs biosynthesized using the leaf extract of *S. bryopteris* exhibited anticancer activity against the SKOV3 and OVCAR-3 human ovarian cancer cell lines. The inclusion of the non-tumorigenic ovarian epithelial cell line IOSE80 further allowed a preliminary comparison between cancer and non-cancer ovarian cellular models, while future studies may include standard reference drugs for comparative purposes. In agreement with these findings, previous studies have reported the cytotoxic potential of green-synthesized AgNPs against different cancer cell lines. For example, Baran et al. (2022) described the synthesis and characterization of AgNPs obtained from *Cicer arietinum* L. green leaf extract and evaluated their antimicrobial and cytotoxic properties. In that study, AgNPs inhibited the growth and proliferation of U118, Caco-2, SKOV3, and HDF cells at concentrations ranging from 25 to 100 μg/mL (Baran et al., 2022). Moreover, another study investigated the interaction between AgNPs and cisplatin in ovarian cancer cell lines, including A2780, SKOV3, and OVCAR-3. The combination of AgNPs with cisplatin showed a synergistic interaction in A2780 and OVCAR-3 cells, supporting the potential use of AgNPs as complementary agents in ovarian cancer treatment (Fahrenholtz et al., 2017). In the present study, the stronger cytotoxicity of *S. bryopteris*-derived AgNPs compared with the extract alone may therefore reflect the combined contribution of the AgNPs core and the extract-derived surface coating. The metallic core may promote silver-ion release and ROS-mediated cytotoxicity, whereas phytochemicals involved in reduction and capping may contribute to AgNPs stabilization and biological activity. Future comparative studies using chemically synthesized or bare AgNPs of comparable size will help clarify the relative contribution of these components. However, the specific contribution of NPs-mediated delivery was not directly investigated and will require further mechanistic studies. The anticancer activity of AgNPs may be associated, at least in part, with their ability to induce ROS production. ROS can react with cellular biomolecules, leading to oxidative stress, mitochondrial dysfunction, DNA damage, and activation of cell death pathways. In this context, the observed ROS generation following AgNP treatment may contribute to the cytotoxic effects detected in SKOV3 cells. However, additional mechanistic studies are required to fully clarify the molecular events underlying AgNP-mediated anticancer activity. In parallel, *in silico* analysis suggested that rhamnetin, a flavonoid present in the aqueous leaf extract of *S. bryopteris*, may represent an interesting scaffold for the development of PARP-1 inhibitors for ovarian cancer treatment. Although rhamnetin and rutin showed favorable predicted interactions with PARP-1 in docking analyses, docking scores and predicted binding affinities alone do not demonstrate biological activity or therapeutic efficacy. Therefore, these computational results should be considered hypothesis-generating and will require further validation through biochemical PARP-1 inhibition assays and target-engagement studies. It should be emphasized that not all phytocompounds included in the docking analysis were experimentally identified in the present extract. Therefore, the computational results should be interpreted as an exploratory, literature-supported screening rather than as direct evidence that these compounds are present in the tested extract or responsible for the observed biological activity. Similarly, the involvement of plant-derived phytoconstituents in AgNP capping and stabilization is supported indirectly by FTIR, DLS, TGA/DTG, and physicochemical characterization, while definitive identification of the surface-associated capping molecules will require dedicated LC-MS/MS, HPLC-based, or surface characterization studies. PARP-1 inhibitors are promising therapeutic agents, particularly in tumors characterized by DNA repair defects, such as BRCA-mutated ovarian cancers. Accordingly, the predicted interaction between rhamnetin and PARP-1 should be considered preliminary and requires validation through dedicated *in vitro* and *in vivo* studies. It should also be noted that the ADMET predictions reported in this study refer to selected phytochemicals identified in *S. bryopteris* extract and should not be directly extrapolated to the green-synthesized AgNP system. Dedicated pharmacokinetics biodistribution, and safety studies will be required to characterize the AgNP formulation itself. Such experiments may provide important insights into the biological relevance of this interaction and support the development of new therapeutic strategies for ovarian cancer. Consistent with this hypothesis, previous evidence has suggested that rhamnetin may interfere with ovarian cancer cell progression. In particular, rhamnetin, a nutraceutical flavonoid, has been reported to inhibit the progression of SKOV3 ovarian cancer cells by affecting cell cycle progression and modulating histone deacetylase 2, a protein involved in chromatin remodeling and the formation of condensed chromatin structures (Bharadwaj et al., 2024). These findings support the potential relevance of rhamnetin as a bioactive compound in ovarian cancer research, although further studies using isolated rhamnetin are necessary to fully define its pharmacological profile, mechanism of action, and therapeutic potential. The clinical use of PARP-1 inhibitors, such as niraparib, is associated with several adverse effects, including fatigue, anemia, thrombocytopenia, nausea, vomiting, and reduced platelet counts. Natural phytochemicals, including terpenoids and polyphenols such as flavonoids, catechins, quercetin, anthocyanins, curcumin, and proanthocyanidins, have attracted attention due to their broad biological activities and generally favorable safety profiles. Similarly, sulfur-containing compounds such as sulforaphane and allicin, derived from plants, fruits, and vegetables, have been investigated for their potential health-promoting properties. Nevertheless, although phytochemicals may represent promising complementary or alternative scaffolds for anticancer drug discovery, their efficacy and safety in ovarian cancer, including BRCA-mutated settings, require rigorous experimental and clinical validation. Before considering phytocompounds and AgNPs for biomedical applications, their safety and biocompatibility must be carefully assessed. In particular, additional studies, including hemocompatibility assays, normal-cell toxicity evaluation, long-term stability studies, and *in vivo* toxicity analyses, are required to define their potential toxicological profile. This is particularly relevant for AgNPs, whose biological effects may depend on size, shape, surface chemistry, capping agents, concentration, and exposure conditions.

AgNPs have gained considerable attention in biomedical research because of their unique physicochemical properties. Among the different methods available for AgNP synthesis, green synthesis is considered an environmentally friendly and sustainable approach compared with conventional chemical and physical methods. In green synthesis, plant-derived phytocompounds, including flavonoids, terpenoids, and alkaloids, can act as reducing, stabilizing, and capping agents. By contrast, chemical synthesis often requires hazardous reducing or stabilizing agents, such as sodium borohydride, lithium aluminium hydride, trisodium citrate, and polyvinyl alcohol. However, both green and chemical synthesis approaches have advantages and limitations, and comparative studies are needed to better understand how the synthesis route affects NPs properties and biomedical performance.

Several critical issues still need to be addressed before translating green-synthesized AgNPs from laboratory-scale production to broader biomedical applications. Scalability, NPs stability, and reproducibility are particularly important parameters. Although phytocompounds present in the leaf extract of *S. bryopteris* may serve as reducing, capping, and stabilizing agents, they may also be affected by environmental conditions such as pH, temperature, and light exposure. These factors can influence AgNP stability and may promote aggregation. The reduced stability observed under strongly acidic conditions, particularly at pH 3, suggests that future preclinical development for oral administration should consider the potential susceptibility of AgNPs to the gastric environment. In this context, protective formulation strategies, including enteric coating or encapsulation into suitable drug delivery systems, may be required to preserve AgNP stability and biological activity. Similarly, although AgNPs could be further explored as platforms for targeted drug delivery or controlled release, these applications were not experimentally investigated in the present study and will require dedicated formulation, release, cellular uptake, and biodistribution studies. Therefore, parameters such as extract concentration and volume, metal salt precursor concentration, incubation time, temperature, and pH should be carefully optimized to improve scalability and reproducibility. Moreover, batch-to-batch variations in plant extracts may affect NPs size, morphology, surface properties, and biological activity, making standardization a major challenge.

*Selaginella bryopteris* is considered a valuable plant system with several reported therapeutic properties. Therefore, the investigation of its phytochemical constituents, including flavonoids, represents a rational approach for the identification of bioactive compounds with potential anticancer relevance. At the same time, conservation of its natural habitats is essential to preserve biodiversity and support future pharmacological and ecological studies. In summary, the present findings support the continued investigation of *S. bryopteris*-derived AgNPs and selected phytochemicals in ovarian cancer models, while their biological relevance and translational potential remain to be established.

## 5 Conclusion

The present study provides preliminary evidence that *S. bryopteris*-derived AgNPs and selected phytoconstituents, particularly rhamnetin, may exert biologically relevant effects in ovarian cancer models. Although these findings are encouraging, they should be interpreted as exploratory and require validation through more detailed mechanistic, pharmacological, toxicological, and *in vivo* studies. These investigations will be essential to clarify the pathways involved, define the safety profile, and determine the actual therapeutic potential of these systems. In addition, the possible application of AgNPs as platforms for targeted delivery or controlled release of anticancer agents deserves further investigation through dedicated studies on formulation, stability, biodistribution, and release kinetics. Overall, the present results provide a rationale for the continued evaluation of *S. bryopteris*-derived nanomaterials and phytochemicals as potential candidates for future nanomedicine-based approaches in ovarian cancer.

## Data Availability

The original contributions presented in the study are included in the article/Supplementary Material, further inquiries can be directed to the corresponding authors.
