# Supplementary material for: Selaginella bryopteris-derived silver nanoparticles against ovarian cancer cells: green synthesis, in vitro anticancer activity, and exploratory in silico analysis
Source: Front Pharmacol. 2026 Aug 11;17:1814120. doi: 10.3389/fphar.2026.1814120 (PMC13503321; doi:10.3389/fphar.2026.1814120)
Supplement: Supplementary file 1 [file DataSheet1.pdf]

Supplementary Material

Selaginella bryopteris-derived silver nanoparticles against ovarian cancer cells: green synthesis, *in vitro* anticancer activity, and exploratory *in silico* analysis

Table of Contents

Supplementary Figure 1 (Figure S1) 2

Supplementary Figure 2 (Figure S2) 2

Supplementary Figure 3 (Figure S3) 2

Supplementary Figure 4 (Figure S4) 3

Supplementary Table 1 (Table S1) 4

Supplementary Table 2 (Table S2) 7

Supplementary Table 3 (Table S3) 8

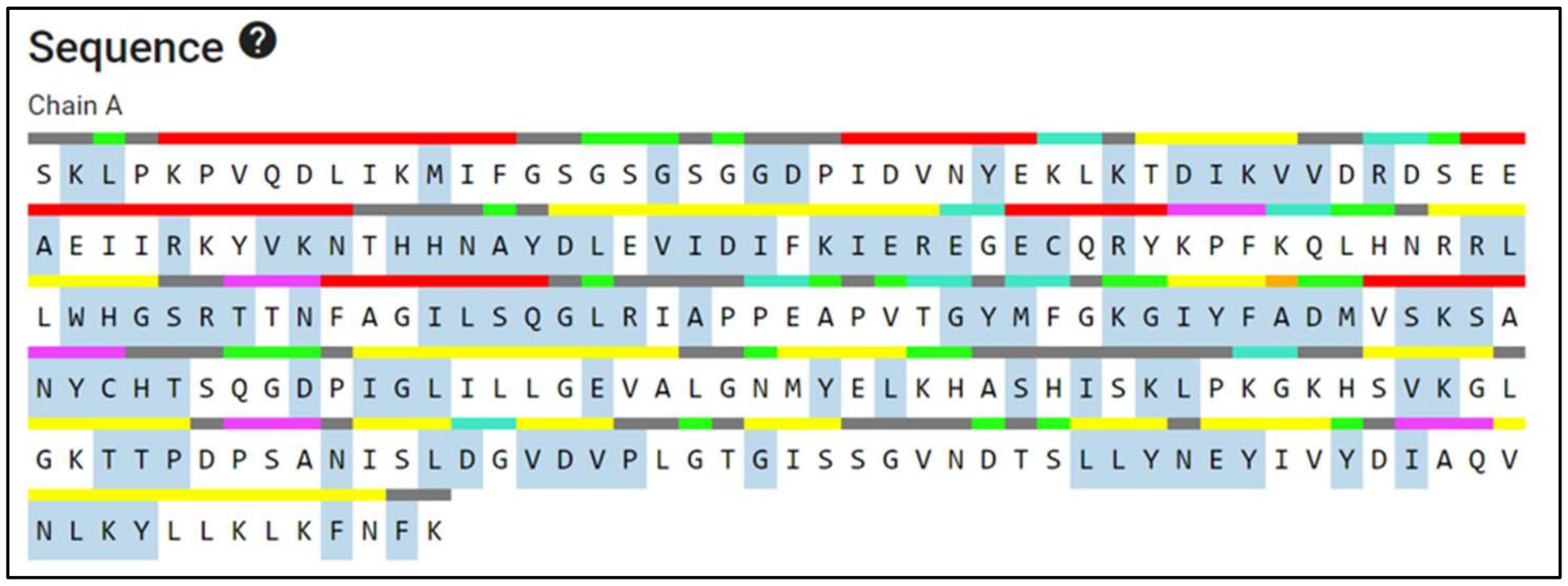

Supplementary Figure 1. Active site prediction by CASTp.

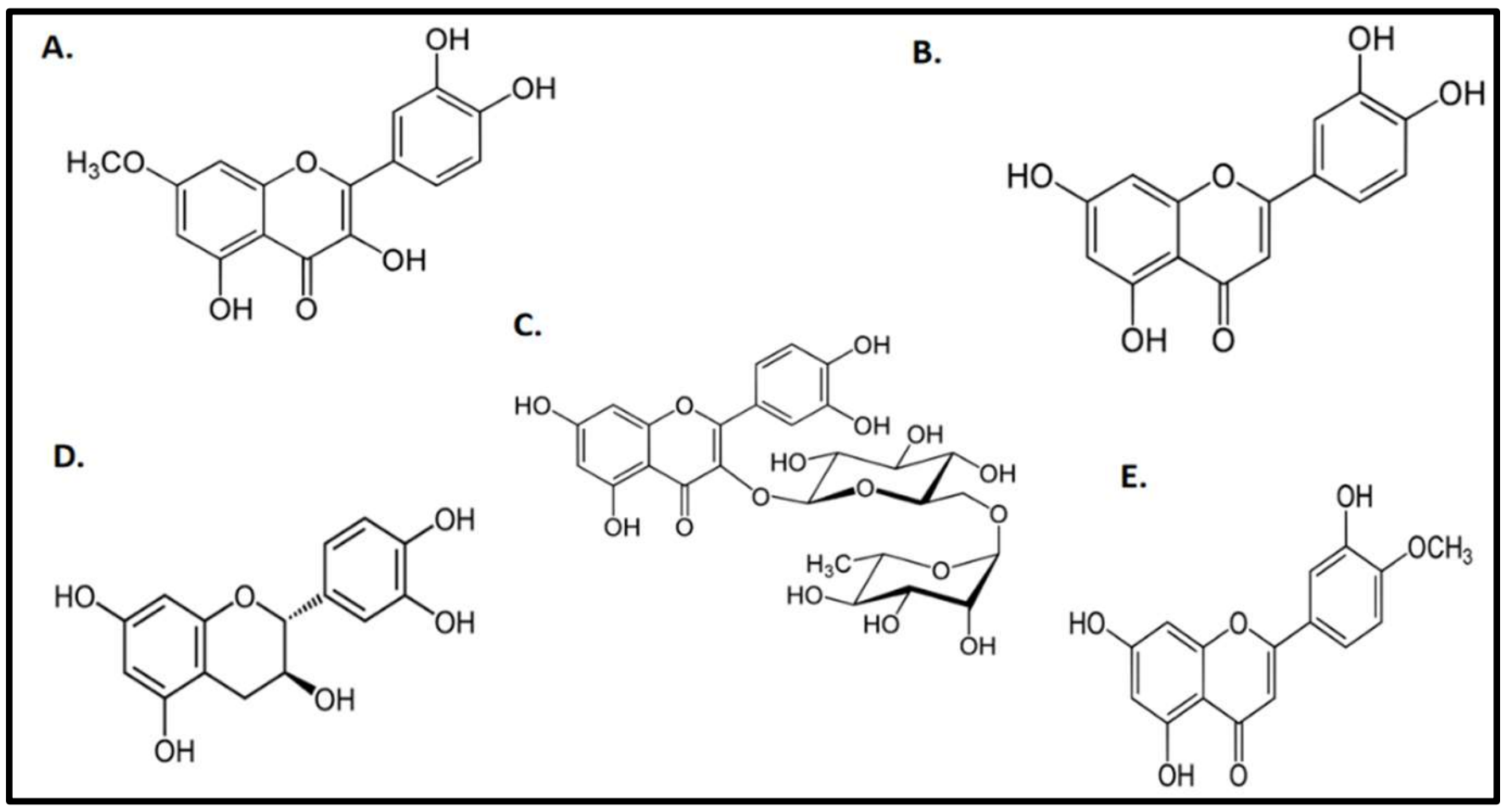

Supplementary Figure 2. Structures of best hit flavonoids against PARP-1 with favorable ADME properties (A) Rhamnetin (B) Luteolin (C) Rutin (D) Catechin (E) Diosmetin.

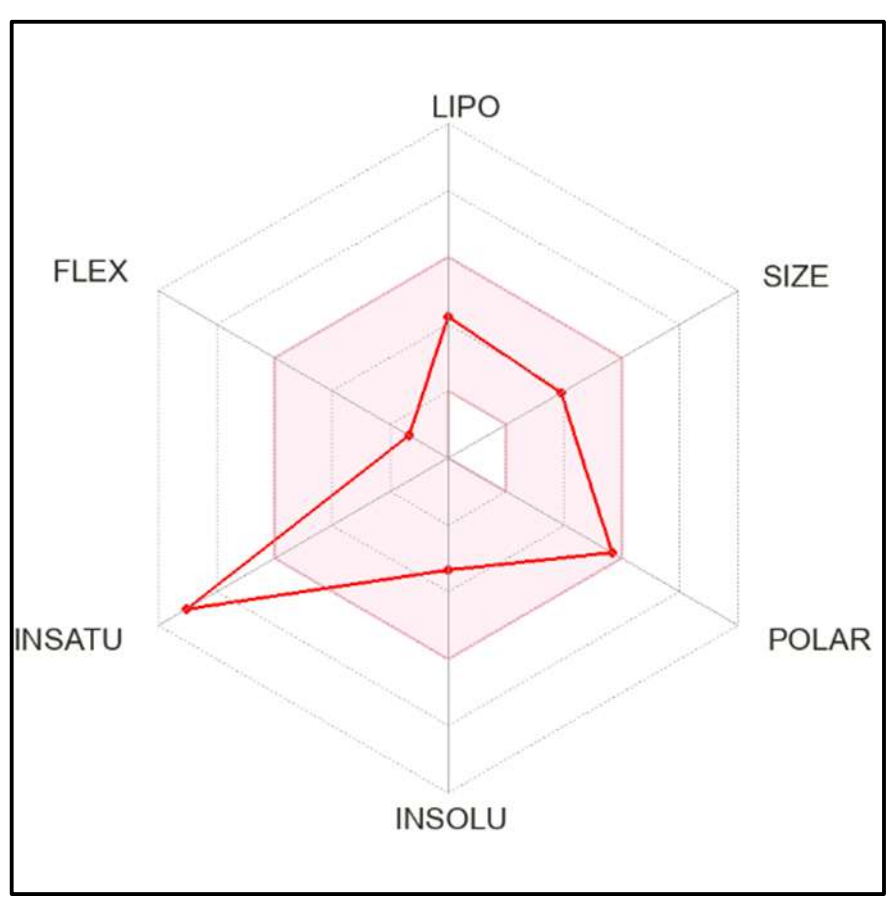

Supplementary Figure 3. Bioavailability radar plot of Rhamnetin; LIPO (lipophilicity); XLOGP3 (-0.33), SIZE (molecular weight 316.26g/mol), POLAR (polarity 120.36 A°), INSOLU (insolubility -3.36), and FLEX (flexibility, number of rotatable bonds 6).

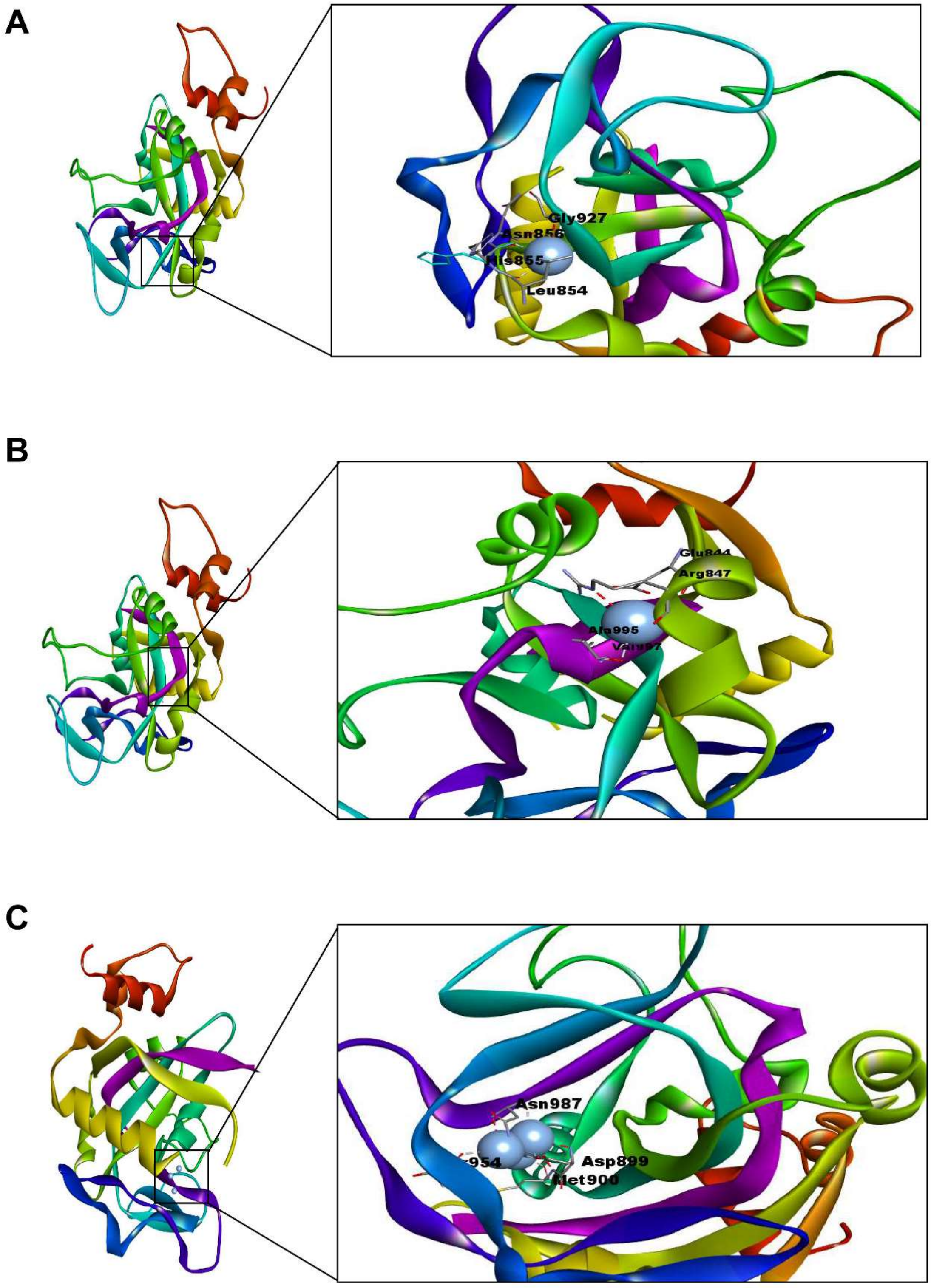

Supplementary Figure 4. Molecular docking interactions of silver atoms (A) Ag, (B) Ag^{+}, and (C) Ag_3 with the amino residues of PARP-1. The binding of the silver to the protein is illustrated on the left side, while the interactions between the amino acid residues and the silver atoms are depicted on the right side.

Supplementary Table 1. GC-MS analysis of leaf extract of *S. bryopteris*.

Peak	R.Time	F.Time	Area	Area%	Height	Name
1	7.772	7.875	442931	0.02	79191	
2	8.095	8.130	265491	0.01	85014	Pyrrolidine
3	8.186	8.400	1884011	0.10	371058	Cyclopentanone
4	8.435	8.530	209553	0.01	66538	2(5H)-Furanone, 5-methyl-
5	9.775	9.915	231749	0.01	32194	Silane, (chloromethyl)ethoxydimethyl-
6	9.969	10.060	452372	0.02	106564	Phenol
7	10.179	10.275	838669	0.04	208319	2-Furanone, 2,5-dihydro-3,5-dimethyl
8	10.585	10.620	1635994	0.08	55015	2-Hydroxy-gamma-butyrolactone
9	11.125	11.200	403559	0.02	113716	2-Cyclopenten-1-one, 2-hydroxy-3-methyl-
10	11.628	11.800	1145380	0.06	201814	Pantolactone
11	12.583	12.655	4616936	0.23	666316	Furaneol
12	12.834	12.970	8162823	0.41	2171259	Phenol, 2-methoxy-
13	13.242	13.380	2268465	0.11	220176	Propanoic acid, 2,2-dimethyl-
14	14.424	14.525	2583706	0.13	787749	Methyl nicotinate
15	15.760	15.865	527332	0.03	113231	Octanoic acid
16	16.357	16.395	210196	0.01	57126	Oxirane, 2-methyl-2-pentyl-
17	16.796	16.970	1841521	0.09	225288	Catechol
18	17.060	17.230	1603289	0.08	325082	4-Vinylphenol
19	17.389	17.700	4254062	0.21	406250	
20	17.960	18.030	328008	0.02	129029	2-Decenal, (E)-
21	18.434	18.505	469329	0.02	104884	Azelaic acid
22	18.657	18.745	709429	0.04	175770	1-Heptanamine, N,N-dimethyl-
23	18.801	18.845	222604	0.01	91611	2-Undecanone
24	19.372	19.515	7914930	0.40	2416988	2-Methoxy-4-vinylphenol
25	19.549	19.620	251722	0.01	80315	2,4-Decadienal, (E,E)-
26	20.035	20.175	777694	0.04	137615	1,3-Dithiolan-2-ylidenecyanamide
27	20.372	20.470	1235340	0.06	351319	Phenol, 2,6-dimethoxy-
28	21.503	21.570	1586169	0.08	610151	1-Tetradecanol
29	21.737	21.790	456795	0.02	183178	Tetradecane
30	23.284	23.345	312575	0.02	101234	Cyclohexane, octyl-
31	24.756	24.805	273384	0.01	62812	Dodecane, 2,6,11-trimethyl-
32	25.688	25.765	573150	0.03	138336	2,4-Di-tert-butylphenol
33	26.065	26.100	703573	0.04	85568	
34	26.960	27.070	1478614	0.07	214648	1,3-Dioxan-4-one, 2-(1-methylethyl)-5-methyl
35	27.676	27.795	4577802	0.23	1109214	Phenol, 4-ethenyl-2,6-dimethoxy-
36	27.891	27.980	483040	0.02	123329	Dodecanoic acid
37	28.562	28.655	4321283	0.22	1638928	1-Hexadecanol
38	28.759	28.810	936075	0.05	395079	Heptadecane
39	28.863	28.905	201705	0.01	95426	1-Dodecanol
40	29.879	29.955	295609	0.01	88200	5-Cyclohexyl-1-pentene
41	30.043	30.080	525122	0.03	218899	Cyclohexane, hexadecyl-
42	30.325	30.380	1830816	0.09	788623	8-Pentadecanone
43	30.733	30.760	209925	0.01	100527	1-Hexadecanol

44	30.874	30.925	809047	0.04	284740	Heptadecane
45	31.145	31.190	416963	0.02	155652	Behenic alcohol
46	31.283	31.335	2321282	0.12	1080861	Methyl tetradecanoate
47	31.607	31.640	350363	0.02	168033	Behenyl chloride
48	31.722	31.770	326489	0.02	130828	
49	31.828	31.880	210056	0.01	98648	Hexadecane, 1-methoxy-13-methyl-
50	32.119	32.235	38045867	1.92	5877476	5-Chlorovaleric acid, heptadecyl ester
51	32.268	32.315	601116	0.03	278809	Tridecane, 3-methylene-
52	32.374	32.440	3481308	0.18	1918848	1-Nonadecene
53	32.473	32.510	820618	0.04	480241	Heneicosane
54	32.539	32.625	511094	0.03	173881	
55	32.718	32.745	265801	0.01	148618	cis-1-Chloro-9-octadecene
56	32.808	32.845	398966	0.02	216214	Isopropyl myristate
57	32.978	33.015	277444	0.01	105659	Hexadecanal
58	33.055	33.075	575127	0.03	343132	2-Pentadecanone, 6,10,14-trimethyl-
59	33.298	33.335	710549	0.04	378502	Phthalic acid, isobutyl cyclohexylmethyl ester
60	33.383	33.425	1442465	0.07	653446	Pentadecanoic acid
61	33.470	33.510	1778908	0.09	1034348	1-Hexacosene
62	33.588	33.620	225536	0.01	122690	
63	33.668	33.705	295694	0.01	125442	n-Nonenylsuccinic anhydride
64	33.839	33.865	342911	0.02	260634	Methyl hexadec-9-enoate
65	33.904	33.930	423259	0.02	249924	7,9-Di-tert-butyl-1-oxaspiro(4,5)deca-6,9-dien
66	34.138	34.165	61686555	3.11	23034765	
67	34.187	34.240	5077530	0.26	2658051	Benzenepropanoic acid, 3,5-bis(1,1-dimethyle
68	34.389	34.405	223637	0.01	85626	
69	34.509	34.550	1222802	0.06	619059	7-Hexadecenoic acid, methyl ester, (Z)-
70	35.311	35.500	545110170	27.52	18563234	
71	36.592	36.625	66201643	3.34	16057670	9,12-Octadecadienoic acid (Z,Z)-, methyl este
72	36.706	36.745	55459308	2.80	15202933	9-Octadecenoic acid, methyl ester, (E)-
73	36.770	36.805	1637207	0.08	917070	9-Octadecenoic acid, methyl ester, (E)-
74	36.869	36.900	529610	0.03	266055	Phytol
75	37.115	37.160	7111219	0.36	2788232	Methyl stearate
76	37.181	37.225	454152	0.02	248027	9,12-Octadecadienoic acid (Z,Z)-, 2-hydroxy-
77	37.290	37.315	541143	0.03	265081	i-Propyl 9-octadecenoate (Z)
78	38.534	38.660	671490676	33.91	13197378	E,E,Z-1,3,12-Nonadecatriene-5,14-diol
79	38.714	38.745	8234543	0.42	3017872	Octadecanoic acid
80	40.399	40.450	8010115	0.40	3434097	Dimethylaminoethyl palmitate
81	40.533	40.565	1131474	0.06	482496	Methyl 5-eicosenoate
82	40.907	40.985	3450156	0.17	1115760	Methyl 18-methylnonadecanoate
83	41.151	41.225	2379459	0.12	767482	cis-13-Eicosenoic acid
84	41.338	41.370	344874	0.02	208924	E,E,Z-1,3,12-Nonadecatriene-5,14-diol
85	41.447	41.530	3720261	0.19	1058764	Eicosanoic acid
86	41.702	41.750	404093	0.02	186785	13-Docosenamide, (Z)-
87	41.934	41.960	739152	0.04	449158	Linoleic acid ethyl ester
88	42.001	42.035	1624751	0.08	618392	
89	42.069	42.100	827010	0.04	487305	9-Octadecenoic acid (Z)-, 2,3-dihydroxypropy
90	42.349	42.375	7338724	0.37	4358188	3-Cyclopentylpropionic acid, 2-dimethylamino
91	42.401	42.435	6375223	0.32	3996316	Bis(2-(Dimethylamino)ethyl) ether
92	42.479	42.510	591302	0.03	328793	Tetracosamethyl-cyclododecasiloxane

93	42.542	42.570	677195	0.03	379979	Oleic Acid, (Z)-, TMS derivative
94	42.846	42.860	29132502	1.47	3980360	.gamma.-Sitosterol
95	42.929	43.005	43604626	2.20	12006845	Thunbergol
96	43.021	43.045	663023	0.03	608494	Methyl 20-methyl-heneicosanoate
97	43.202	43.340	4225126	0.21	844208	
98	43.412	43.450	431222	0.02	218070	RT:43.410
99	43.483	43.570	633326	0.03	233027	13-Docosenamide, (Z)-
100	43.630	43.700	1078267	0.05	431905	Tetracosamethyl-cyclododecasiloxane
101	43.764	43.795	580535	0.03	289719	Eicosanoic acid, 2-[(1-oxohexadecyl)oxy]-1-[[
102	43.909	44.065	7879088	0.40	1053308	
103	44.119	44.175	381868	0.02	129351	1-Cyclohexyldimethylsilyloxybutane
104	44.408	44.445	613777	0.03	306803	1,1,1,3,3,5,5,7,7,9,9,11,11,13,13,15,15,17,17,
105	44.690	44.820	165360305	8.35	18018927	9-Octadecenoic acid (Z)-, 2,3-dihydroxypropy
106	44.865	44.880	3155712	0.16	1549447	Octadecanoic acid, 2,3-dihydroxypropyl ester
107	44.912	45.030	7402920	0.37	3054218	Tetracosanoic acid, methyl ester
108	45.384	45.460	15783530	0.80	2171997	9,19-Cyclolanost-24-en-3-ol, acetate, (3.beta.)
109	45.524	45.635	24261618	1.23	7771490	13-Docosenamide, (Z)-
110	45.836	45.890	7537147	0.38	3083163	Squalene
111	46.027	46.065	569151	0.03	244033	Methyl 22-methyl-tetracosanoate
112	46.132	46.190	580428	0.03	178934	Phenol, 2,4-bis(1,1-dimethylethyl)-, phosphite
113	46.298	46.350	554647	0.03	240202	Tetracosamethyl-cyclododecasiloxane
114	46.467	46.545	833283	0.04	295640	Methyl 2-hydroxy-tetracosanoate
115	46.702	46.835	11481483	0.58	1518990	9,19-Cyclo-25,26-epoxyergostan-3-ol, 4,4,14-
116	46.904	46.970	301017	0.02	103065	Tetrapentacontane
117	47.268	47.400	26002242	1.31	2390363	.gamma.-Sitostenone
118	47.542	47.555	10904320	0.55	2185724	9,19-Cyclolanostan-3-ol, 24-methylene-, aceta
119	47.583	47.735	11198294	0.57	2278158	
120	47.821	47.890	1010466	0.05	272542	1H-Imidazole, 1-(1-oxooctadecyl)-
121	48.236	48.385	1999843	0.10	464554	Palmitic acid vinyl ester
122	48.521	48.580	360653	0.02	131722	2,2,4-Trimethyl-3-(3,8,12,16-tetramethyl-hept
123	48.607	48.680	293353	0.01	89845	Ergost-5-en-3-ol, acetate, (3.beta.,24R)-
124	49.348	49.445	2878397	0.15	717979	.gamma.-Tocopherol
125	49.508	49.570	411686	0.02	117979	(R)-2,8-Dimethyl-2-((3E,7E)-4,8,12-trimethyl
126	49.784	49.850	641528	0.03	148975	Cholesta-4,6-dien-3-ol, (3.beta.)-
127	49.922	50.030	476473	0.02	101253	
128	50.180	50.295	667322	0.03	155447	.beta.-Sitosterol acetate
129	50.380	50.450	371703	0.02	102524	Tetracosamethyl-cyclododecasiloxane
130	50.746	50.810	2265923	0.11	308964	E,E,Z-1,3,12-Nonadecatriene-5,14-diol
131	50.873	50.935	337898	0.02	93457	.alpha.-Tocopherol-.beta.-D-mannoside
132	50.978	51.035	281632	0.01	93137	Octacosanoic acid, methyl ester
133	51.172	51.325	1829738	0.09	227839	
134	51.755	51.945	10045036	0.51	1465623	(R)-2,7,8-Trimethyl-2-((3E,7E)-4,8,12-trimeth
			1980498687	100.00	214491982	

Supplementary Table 2. Binding energies of selected phytochemicals presents in aqueous extract of *Selaginella bryopteris* against PARP-1.

**Class**	**Name of Compound**	**Binding Energy (kcal/mol)**
**Alkaloids**	8-Hydroxyquinoline	-7.68
	Guvacoline	-7.13
**Flavonoids**	Rutin	-16.13
	Rhamnetin	-11.56
	Catechin	-11.40
	Luteolin	-11.19
	Diosmetin	-10.83
	Kaempferol	-10.61
	Myo-inositol	-9.00
**Biflavonoid**	2,3-Dihydroamentoflavone	-17.31
	Tetrahydroamentoflavone	-16.78
	Bilobetin	-16.73
	Amentoflavone	-16.67
	Sequoiaflavone	-15.93
	Heveaflavone	-15.81
	Sciadopitysin	-15.73
	Lanaroflavone	-14.79
	2,3-Dihydrohinokiflavone	-13.77
	2",3"-dihydroamentoflavone	-13.52
**Reference Ligand**	Olaparib	-15.15

Supplementary Table 3. ADMET profile and drug-likeness of the top-ranked phytocompounds.

	Molecular descriptor	2,3-Dihydroamentoflavone	Tetrahydroamentoflavone	Bilobetin	Amentoflavone	Rutin	Rhamnetin	Olaparib
**PhysChem parameters**								
	Molecular formula	$C_{30}H_{20}O_{10}$	$C_{30}H_{22}O_{10}$	$C_{31}H_{20}O_{10}$	$C_{30}H_{18}O_{10}$	$C_{27}H_{30}O_{16}$	$C_{16}H_{12}O_7$	$C_{24}H_{23}FN_4O_3$
	No of rotatable bonds	3	3	4	3	6	2	6
	Molar refractivity	144.55	142.13	151.44	146.97	141.83	82.50	125.21
	Fraction Csp3	0.07	0.13	0.03	0.00	0.44	0.06	0.33
	No. of heavy atoms	40	40	41	40	43	23	32
	No. of aromatic heavy atoms	28	24	32	32	16	16	16
	TPSA (Å^2)	177.89	173.98	170.80	181.80	269.43	120.36	86.37
**Lipophilicity**								
	Log $P_{o/w}$ (iLOGP)	2.82	2.55	3.39	3.06	1.58	2.23	2.84
	Log $P_{o/w}$ (XLOGP3)	4.76	4.49	5.36	5.04	-0.33	1.87	1.90
	Log $P_{o/w}$ (WLOGP)	4.74	4.35	5.44	5.13	-1.69	2.29	1.94
	Log $P_{o/w}$ (MLOGP)	0.41	0.58	0.44	0.25	-3.89	-0.31	3.09
	Log $P_{o/w}$ (SILICOS-IT)	4.15	3.68	5.16	4.61	-2.11	2.06	4.14
	Consensus Log $P_{o/w}$	3.38	3.13	3.96	3.62	-1.29	1.63	2.78
**PK**								
	GI-absorption	Low	Low	Low	Low	Low	High	High
	BBB permeant	No	No	No	No	No	No	No
	P-gp substrate	No	No	No	No	Yes	No	Yes
	CYP2C19 inhibitor	No	No	No	No	No	Yes	No
	CYP2C19 inhibitor	No	No	No	No	No	No	Yes
	CYP2C9 inhibitor	Yes	Yes	Yes	No	No	No	Yes
	CYP2D6 inhibitor	No	No	No	No	No	Yes	Yes
	CYP3A4 inhibitor	No	No	No	No	No	Yes	Yes
	Log Kp (skin permeation, cm/s)	-6.22	-6.42	-5.86	-6.01	-10.26	-6.90	7.60
**Drug likeliness**								
	Lipinski	No 2 violations	No 2 violations	Yes 1 violation	No 2 violations	No 3 violations	Yes 0 violation	Yes 0 violation
	Ghose	No 2 violations	No 2 violations	No 2 violations	No 2 violations	No 4 violations	Yes	Yes
	Veber	No 1 violation	No 1 violation	No 1 violation	No 1 violation	No 1 violation	Yes	Yes
	Egan	No 1 violation	No 1 violation	No 1 violation	No 1 violation	No 1 violation	Yes	Yes
	Muegge	No 2 violations	No 2 violations	No 2 violations	No 3 violations	No 4 violations	Yes	Yes
	Bioavailability score	0.17	0.17	0.55	0.17	0.17	0.55	0.55
